# Transient juvenile hypoglycemia in GH insensitive Laron syndrome pigs is associated with insulin hypersensitivity

**DOI:** 10.1016/j.molmet.2025.102273

**Published:** 2025-10-20

**Authors:** Arne Hinrichs, Kalliopi Pafili, Gencer Sancar, Laeticia Laane, Silja Zettler, Malek Torgeman, Barbara Kessler, Judith Leonie Nono, Sonja Kunz, Birgit Rathkolb, Cristina Barosa, Cornelia Prehn, Alexander Cecil, Simone Renner, Elisabeth Kemter, Sabine Kahl, Julia Szendroedi, Martin Bidlingmaier, John Griffith Jones, Martin Hrabĕ de Angelis, Michael Roden, Eckhard Wolf

**Affiliations:** 1 Chair for Molecular Animal Breeding and Biotechnology, Gene Center and Department of Veterinary Sciences, LMU Munich, Munich, Germany; 2 Center for Innovative Medical Models (CiMM), LMU Munich, Oberschleissheim, Germany; 3 Department of Endocrinology and Diabetology, Medical Faculty and University Hospital Düsseldorf, Heinrich-Heine-University Düsseldorf, Düsseldorf, Germany; 4 Institute for Clinical Diabetology, German Diabetes Center, Leibniz Center for Diabetes Research at Heinrich-Heine-University Düsseldorf, Düsseldorf, Germany; 5 German Center for Diabetes Research (DZD), Neuherberg, Germany; 6 Institute for Diabetes Research and Metabolic Diseases of the Helmholtz Center Munich, Tübingen, Germany; 7 Department of Internal Medicine IV, Division of Diabetology, Endocrinology and Nephrology, University Hospital of Tübingen, Tübingen, Germany; 8 Endocrine Laboratory, Medizinische Klinik und Poliklinik IV, Klinikum der Universität München, Munich, Germany; 9 Institute of Experimental Genetics, German Mouse Clinic (GMC), Helmholtz Center Munich, Neuherberg, Germany; 10 Center for Neurosciences and Cell Biology, UC Biotech, Cantanhede, Portugal; 11 Metabolomics and Proteomics Core, Helmholtz Center Munich, Neuherberg, Germany; 12 Department of Endocrinology, Diabetology, Metabolism and Clinical Chemistry, Heidelberg University Hospital, Heidelberg, Germany; 13 Joint Heidelberg-IDC translational Diabetes Program, Helmholtz Center Munich, Neuherberg, Germany; 14 Portuguese Diabetes Association, Lisbon, Portugal; 15 Chair of Experimental Genetics, School of Life Science Weihenstephan, Technical University Munich, Freising, Germany; 16 Interfaculty Center for Endocrine and Cardiovascular Disease Network Modelling and Clinical Transfer (ICONLMU), LMU Munich, Munich, Germany

**Keywords:** GH insensitivity, Hypoglycemia, Insulin sensitivity, Large animal model, Glucose metabolism, Beta-oxidation

## Abstract

**Background and aims:**

Fasting hypoglycemia has clinical implications for children with growth hormone (GH)-insensitivity syndrome. This study investigates the pathophysiology of juvenile hypoglycemia in a large animal model for GH receptor (GHR) deficiency (the *GHR*-KO pig) and elucidates mechanisms underlying the transition to normoglycemia in adulthood.

**Methods:**

Insulin sensitivity was assessed in juvenile and adult *GHR*-KO pigs and wild-type (WT) controls via hyperinsulinemic-euglycemic clamp (HEC) tests. Glucose turnover was measured using D-[6,6-^2^H_2_] glucose and ^2^H_2_O. Clinical chemical and targeted metabolomics parameters in blood serum were correlated with qPCR and western blot analyses of liver and adipose tissue.

**Results:**

*GHR*-KO pigs showed increased insulin sensitivity (p = 0.0019), especially at young age (M-value +34% vs. WT), insignificantly reduced insulin levels, and reduced endogenous glucose production (p = 0.0007), leading to fasting hypoglycemia with depleted liver glycogen, elevated β-hydroxybutyrate, but no increase in NEFA levels. Low hormone-sensitive lipase phosphorylation in adipose tissue suggested impaired lipolysis in young *GHR*-KO pigs. Metabolomics indicated enhanced fatty acid beta-oxidation and use of glucogenic amino acids, likely serving as compensatory pathways to maintain energy homeostasis. In adulthood, insulin sensitivity remained elevated but less pronounced (M-value +20%), while insulin levels were significantly reduced, enabling normoglycemia and improved NEFA availability. Increased fat mass, but not sex hormones, appeared key to this metabolic transition, as early castration had no effect.

**Conclusions:**

Juvenile hypoglycemia in GH insensitivity results from excessive insulin sensitivity, reduced glucose production, and impaired lipolysis. Normoglycemia in adulthood emerges through increased adiposity and moderated insulin sensitivity, independently of sex hormones. These findings elucidate the age-dependent metabolic adaptations in GH insensitivity.

## Highlights

• *GHR*-KO pigs show transient juvenile hypoglycemia, resembling human Laron syndrome.
• Young *GHR*-KO pigs display insulin hypersensitivity, reduced EGP, increased beta-oxidation and ketosis.
• Adult *GHR*-KO pigs accumulate adipose tissue, are less insulin sensitive and normoglycemic.
• The transition to normoglycemia does not depend on sex hormones.

## Graphical abstract

**Figure:**
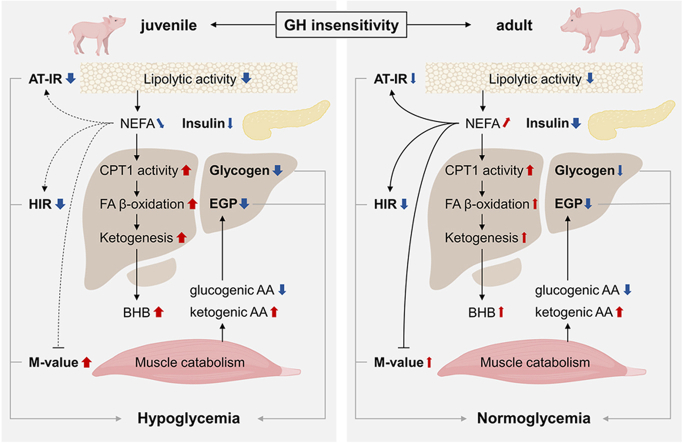

## 1 Introduction

Growth hormone (GH) intrinsically regulates growth and metabolism. Particularly under fasting conditions, elevated GH levels stimulate lipolysis, releasing free fatty acids [1]. Antagonistic effects of GH on insulin action reduce glucose uptake and stimulate endogenous glucose production [2].

GH insensitivity due to growth hormone receptor (GHR) deficiency (GHRD, human Laron syndrome, LS) is characterized by postnatal growth retardation and alterations in body composition [3,4]. LS patients show an increased accumulation of adipose tissue and a reduction in lean/muscle mass. Paradoxically, increased insulin sensitivity despite obesity is described for a cohort of people with LS [5] and is thought to protect against the development of diabetes [6].

At infancy, hypoglycemia upon fasting is reported for LS patients and GH-deficient children in general [7] and has clinical implications, leading to heavy sweating, pallor, headache, seizures, and even loss of consciousness [8]. Proposed mechanisms include enhanced insulin sensitivity and impaired endogenous glucose production (EGP), but direct evidence across developmental stages remains limited [9]. The resolution of hypoglycemia in adulthood has been attributed to pubertal changes [7], yet the role of sex hormones in this transition is underexplored and remains unclear. A comprehensive investigation into the age-dependent metabolic adaptations in GHRD is still lacking and essential to understanding GH insensitivity-associated glucose dysregulation.

As pigs closely resemble human anatomy, physiology and metabolism in particular [10], we studied the metabolic alterations due to GH insensitivity in a large animal model for Laron syndrome (the *GHR*-KO pig [11]) in an age-dependent manner. Previous studies already revealed that *GHR*-KO pigs closely resemble the hallmarks of GH insensitivity such as endocrine disruptions, postnatal growth retardation, altered body composition, juvenile hypoglycemia, decreased insulin secretory capacity, and alterations in metabolic pathways in the liver [[11], [12], [13], [14], [15], [16]]. The current investigations include the assessment of body composition, insulin sensitivity using hyperinsulinemic-euglycemic clamp (HEC) tests, targeted metabolomics, and molecular analyses of liver and adipose tissue. Glucose turnover was assessed with D-[6,6-^2^H_2_] glucose and ^2^H_2_O. We investigated age-dependent differences by comparing young, hypoglycemic *GHR*-KO pigs with adult normoglycemic *GHR*-KO pigs and age-matched controls as well as adult *GHR*-KO pigs neutered at young age. We hypothesized that the increased insulin sensitivity is the main driver of fasting hypoglycemia in GH insensitivity, limiting metabolic counter-regulatory mechanisms such as hypoglycemia-induced EGP promotion and lipolysis. Further, we elucidate whether the transition towards normoglycemia at adult age is mediated by sex hormones.

## 2 Material and methods

### 2.1 Animals and study design

All animal procedures were approved by the responsible animal welfare authority (Regierung von Oberbayern; permission ROB 55.2–2532.Vet_02-17-136) and performed according to the German Animal Welfare Act and Directive 2010/63/EU on the protection of animals used for scientific purposes.

In total, 30 pigs were used in the current study. The animals were grouped according to genotype, age, and sex. The collective of young, prepubertal animals contained 5 female *GHR*-KO and 5 female wild-type (WT) pigs aged 3 months. For the adult age group, 7–8.5 months old pigs were selected because they were sexually mature and *GHR*-KO pigs exhibit normoglycemia at this stage [11]. The collective of adult animals contained 7 WT pigs (3 males, 4 females) and 7 *GHR*-KO pigs (3 males, 4 females). Additionally, 3 male and 3 female *GHR*-KO pigs were neutered at 3 months of age by surgical removal of testes or ovaries. This cohort of early castrated *GHR*-KO pigs was raised to adult age and compared with the 7 intact *GHR*-KO pigs.

*GHR*-KO pigs carrying a frameshift mutation in *GHR* exon 3 [11] were propagated by heterozygous × heterozygous mating enabling the comparison to WT littermate controls.

### 2.2 Metabolic studies

All animals were equipped with central venous catheters in the internal jugular vein as described previously [17] to maintain constant infusion of tracers, glucose and insulin. Furthermore, the internal carotid artery was catheterized to ensure stress-free withdrawal of repeated blood samples. The surgical procedures were performed one week prior to the experiments. After an overnight fasting period (16 h), the animals received a priming infusion of ^2^H_2_O (Sigma–Aldrich, St Louis, USA) within 30 min to assess the contributions of gluconeogenesis to glucose production from the ratio of the ^2^H enrichments in carbon 5 over carbon 2 in blood glucose [[18], [19], [20]]. ^2^H enrichments were measured using LS-MS after derivatization of glucose to acetaminophen glucuronide. The initial dose of 0.5 g ^2^H_2_O per kg body weight was doubled in subsequent experiments and the equilibration time before arterial blood sampling was increased from 3 to 4 h to achieve an enrichment of 0.5 % ^2^H_2_O in body water.

To assess whole-body glucose disposal and endogenous glucose production (EGP), a primed (10 min) infusion of 14 mg/kg/h deuterated glucose (D-[6,6-^2^H_2_] glucose) was administered beginning with the ^2^H_2_O tracer infusion and maintained with an infusion rate of 2 mg/kg/h until the completion of the clamp procedure [21]. The hyperinsulinemic-euglycemic clamp (HEC) was initiated with a priming insulin dose of 10 mIU per kg body weight per minute (Insuman® Rapid, Sanofi-Aventis, Frankfurt am Main, Germany) for the first 10 min, followed by a continuous infusion of 1.5 mIU per kg body weight per minute for 3 h [17,22]. Arterial blood samples were collected at 5-minute intervals for plasma glucose measurement. Simultaneously, an intravenous infusion of 20 % glucose, containing 2.0 % deuterated glucose was adjusted dynamically to maintain a target blood glucose concentration of 90 mg/dL (5 mmol/L). Insulin-stimulated whole-body glucose disposal (M-value) was calculated as previously described during the last 30 min of the clamp, including extracellular space correction [23].

Endogenous glucose production (EGP) during the hyperinsulinemic-euglycemic clamp (HEC) was fully suppressed in both groups (data not shown). Basal EGP was measured under fasting conditions and was calculated using glucose turnover rates derived from isotope enrichment data applying the Steele's non-steady-state equation [23,24]. Tracer-derived atom percent enrichment (APE) of deuterated glucose in plasma was measured using gas chromatography-mass spectrometry (GC–MS) [25].

**Figure S1:**
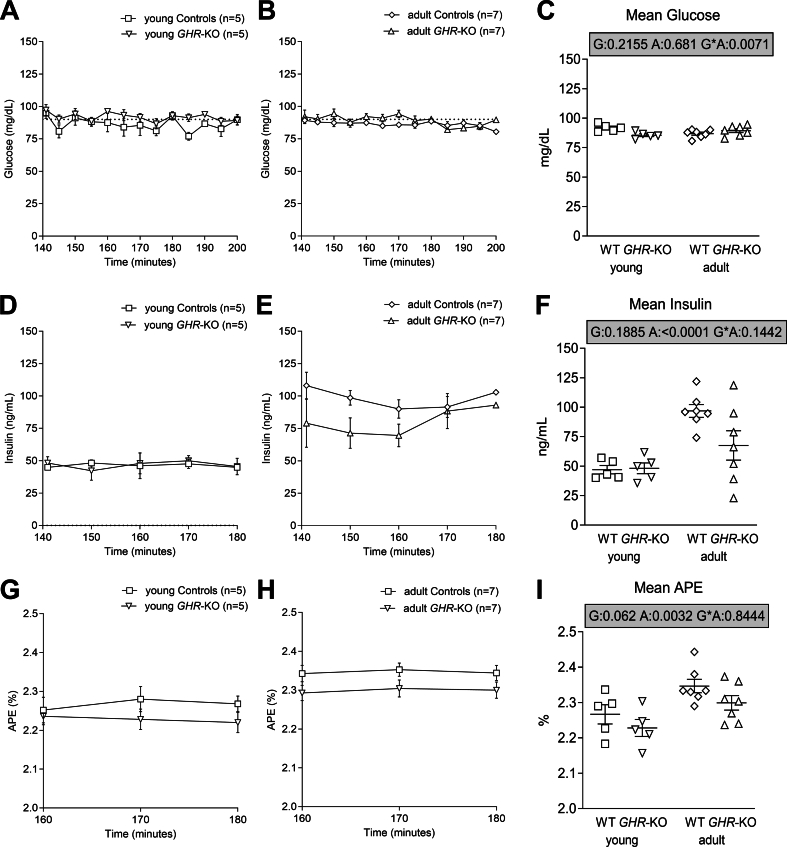
Glucose (A-C), insulin (D-F) levels and APE (G-I) in control and *GHR*-KO pigs during HEC.

Adipose tissue (AT) insulin sensitivity was assessed based on the suppression of non-esterified fatty acid (NEFA) levels. Fasting NEFA levels were measured before insulin infusion, and steady-state NEFA levels were determined during the clamp. NEFA suppression was calculated as: [%] = 100 ∗ (1 – (NEFA_HEC_/NEFA_fasting_)). Glucose and insulin levels as well as APE of blood glucose during clamp steady state are shown in Fig. S1. In addition, tissue-specific insulin resistance was calculated for the basal, fasting state [26] using the following equations:

Fasting hepatic insulin resistance (HIR) = EGP_fasting_ [mg/kg∗min] ∗ fasting insulin [μIU/mL].

Fasting adipose tissue insulin resistance (AT IR) = plasma NEFA [mmol/L] ∗ fasting insulin [μIU/mL].

### 2.3 Metabolite and hormone assays

IGF1 concentrations were measured using the iSYS automated chemiluminescent IGF1 assay (Immunodiagnostic Systems) as described previously [27]. Commercially available assay kits were applied to measure serum glucagon (10-1281-01, Mercodia), insulin [11], and C peptide (10-1256-01, Mercodia) levels. Plasma was isolated by centrifugation from blood collected in EDTA-coated tubes for the measurement of plasma non-esterified fatty acids (NEFA), triglycerides, cholesterol and β-hydroxybutyrate (BHB; Cayman Chemicals, catalog no: Cay700740) as described previously [28]. Steroid measurements by LC–MS/MS were performed using a commercially available kit (Chromsystems, Darmstadt, Germany) according to the manufacturer's instructions as described previously [29]. Liver glycogen content was measured using a commercial calorimetric kit (ab65620, Abcam). Plasma glycerol levels were measured using the Free Glycerol Reagent (Catalog Number F6428, Sigma–Aldrich) following the manufacturer's instructions.

### 2.4 Targeted metabolomics measurements

Targeted metabolomics measurements of the pig serum samples were performed using liquid chromatography- and flow injection-electrospray ionization-tandem mass spectrometry (LC- and FIA-ESI-MS/MS) and the Absolute*IDQ*™ p180 Kit (BIOCRATES Life Sciences AG, Innsbruck, Austria). The complete assay procedures, sample preparation techniques, and detailed metabolite nomenclature have been previously published [30].

Mass spectrometric analyses were done on an API4000 triple quadrupole system (SCIEX Deutschland GmbH, Darmstadt, Germany) equipped with a 1260 Series HPLC (Agilent Technologies Deutschland GmbH, Böblingen, Germany) and an HTC-xc PAL auto sampler (CTC Analytics, Zwingen, Switzerland) controlled by the software Analyst 1.6.2. For the LC-part, compounds were identified and quantified based on scheduled multiple reaction monitoring measurements (sMRM), for the FIA-part on MRM. Data evaluation for quantification of metabolite concentrations and quality assessment were performed with the Web*IDQ*™ software package, which is an integral part of the Absolute*IDQ*™ kit. Metabolite concentrations were calculated using internal standards and reported in μmol/L (μM). Metabolome data were analyzed using the Metaboanalyst software 6.0 [31]. Values were normalized by sum, data were normalized by log 10 transformation and scaled by Pareto.

### 2.5 Necropsy

Necropsy was performed after a resting time of 1 week after HEC and an overnight fasting period (16 h). Pigs were anesthetized by intravenous injection of ketamine (Ursotamin®, Serumwerk Bernburg) and xylazine (Xylazin 2%, Serumwerk Bernburg) followed by fentanyl (Fentadon®, Dechra) application. Samples from liver, visceral and subcutaneous adipose tissues were collected subsequent to exsanguination as described previously [32] and immediately frozen on dry ice and stored at −80 °C for molecular profiling. In accordance with a previous study in *GHR-*KO pigs [11], the ratio of subcutaneous adipose tissue to longissimus lumborum muscle thickness was measured at the level of the last rib.

### 2.6 Western blot analysis of phosphoenolpyruvate carboxykinase 1 and hormone sensitive lipase activity

Concentrations and phosphorylation states of phosphoenolpyruvate carboxykinase 1 (PCK1) in liver and hormone sensitive lipase (HSL) adipose tissues were evaluated by western blot analyses as described previously [11] and outlined in **supplementary material 1**. Band intensities were quantified using the ImageJ software package [33].

### 2.7 RT-qPCR analysis of candidate transcripts in subcutaneous adipose tissue

Frozen subcutaneous adipose tissue samples (100–200 mg) were lysed with 1 mL QIAzol lysis reagent with bead beater in bead tubes (MN, Type F) for 1 min. After adding 250 μL chloroform, tubes were centrifuged at 4 °C at 12,000×*g* to collect RNA upper phase. Isopropanol 1:1 in volume was mixed with the supernatant and RNA purification was performed with RNeasy Kit with on column DNase digestion step (Qiagen, catalog no: 74106). cDNA was prepared with Transcriptor cDNA Synthesis Kit (Roche, cat no: 4897030001) and qPCR was performed with PowerTrack™ SYBR Green Master Mix (Life Technologies, catalog no: A46112). Primers used for qPCR in subcutaneous adipose tissue are listed in Table S1. The expression pattern was visualized using www.heatmapper.ca [34].

### 2.8 Statistical analysis

Data were analyzed using PROC GLM (SAS 8.2) taking the effects of genotype (=group; *GHR*-KO, WT), age (young, adult), and the interaction of group∗age into account. Differences between the sexes and the effect of castration in adult *GHR*-KO pigs were evaluated comparing adult animals using PROC GLM taking the effects of group, sex and the interaction of group∗sex into account. Least squares means (LSMs) and standard errors (SEs) of LSMs were calculated for groups and compared using Student t-tests. Correction for multiple testing was performed with the Bonferroni method using the ADJUST = BON statement. Western immunoblot data were evaluated for significant differences between *GHR*-KO and WT pigs using Mann–Whitney U test. The effects of early castration on sex hormone levels at adult age were evaluated by student's t test using GraphPad PRISM Version 5.04.

## 3 Results

### 3.1 *GHR*-KO pigs display juvenile hypoglycaemia, growth retardation and increased accumulation of adipose tissue

**Figure 1:**
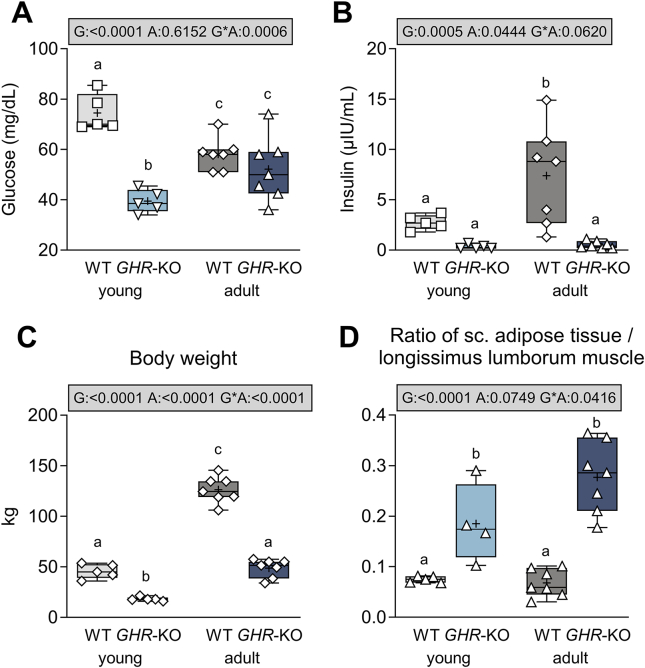
Fasting serum glucose and insulin levels, body weight, and body composition of young and adult *GHR*-KO pigs and WT controls. (**A**) Fasting serum glucose; (**B**) Fasting serum insulin. (**C**) Body weight. (**D**) Fat-to-muscle ratio (thickness of overlaying subcutaneous adipose tissue/longissimus lumborum muscle height). The box plots show median, interquartile range (box) and extremes (whiskers). The mean is marked as “+”. Results of analysis of variance are indicated: G: p-value of the effect of Group; A: p-value of the effect of Age; G∗A: p-value of the interaction Group∗Age. Means with different superscript letters are significantly different (p < 0.05).

Fasting blood glucose levels in young *GHR*-KO pigs were almost halved (−47%) compared to age-matched WT controls (39.5 ± 3.9 mg/dL in *GHR*-KO vs. 74.5 ± 3.9 mg/dL in WT pigs; p < 0.0001, Figure 1A, Table S2). However, at adult age, fasting glucose levels in *GHR*-KO pigs were similar to those in adult WT controls (52.1 ± 3.3 mg/dL in *GHR*-KO vs. 58.1 ± 3.3 mg/dL in WT pigs; p = 0.2099). Serum insulin as well as C-peptide levels were generally lower in *GHR*-KO pigs (p = 0.0005 and p = 0.0015 for the effect of group, Figure 1B, Table S2). The reduction of insulin levels in *GHR*-KO pigs was more pronounced in the adult (p = 0.0001) than in the young age group (p = 0.1734; Figure 1B).

Serum IGF1 levels were reduced by 95% in young and by 90% in adult *GHR*-KO pigs compared to age-matched WT controls (p < 0.0001, Table 1).

**Table 1:** GH insensitivity related alterations in body composition of *GHR*-KO vs. WT pigs. Mean ± SEM; results of analysis of variance.

Parameter	young WT	young *GHR*-KO	adult WT	adult *GHR*-KO	Group	Age	Group∗Age
IGF1 (ng/mL)	344.1 ± 21.0	17.0 ± 21.0	202.1 ± 17.8	19.5 ± 20.2	**<0.0001**	**0.0051**	**0.0039**
Body weight (kg)	45.6 ± 4.2	18.2 ± 4.2	126.2 ± 3.6	48.7 ± 3.5	**<0.0001**	**<0.0001**	**<0.0001**
Subcutaneous fat (cm)	0.4 ± 0.02	0.5 ± 0.1	0.5 ± 0.07	1.2 ± 0.1	**0.0004**	**0.0022**	**0.0160**
Muscle (m. long. lumb.) (cm)	5.1 ± 0.3	3.0 ± 0.3	6.9 ± 0.08	4.2 ± 0.2	**<0.0001**	**<0.0001**	0.2372
Fat/muscle ratio	0.07 ± 0.003	0.19 ± 0.04	0.07 ± 0.01	0.28 ± 0.02	**<0.0001**	0.0749	**0.0416**

Already at young age, the body weight of *GHR*-KO pigs was reduced in comparison with age-matched WT controls (18.2 ± 4.2 kg vs. 45.6 ± 4.2 kg; p < 0.0001, Figure 1C). This 60% reduction of body weight persisted at adult age (48.7 ± 3.5 kg in *GHR*-KO vs. 126.2 ± 3.3 kg in WT pigs; p < 0.0001).

**Figure S2:**
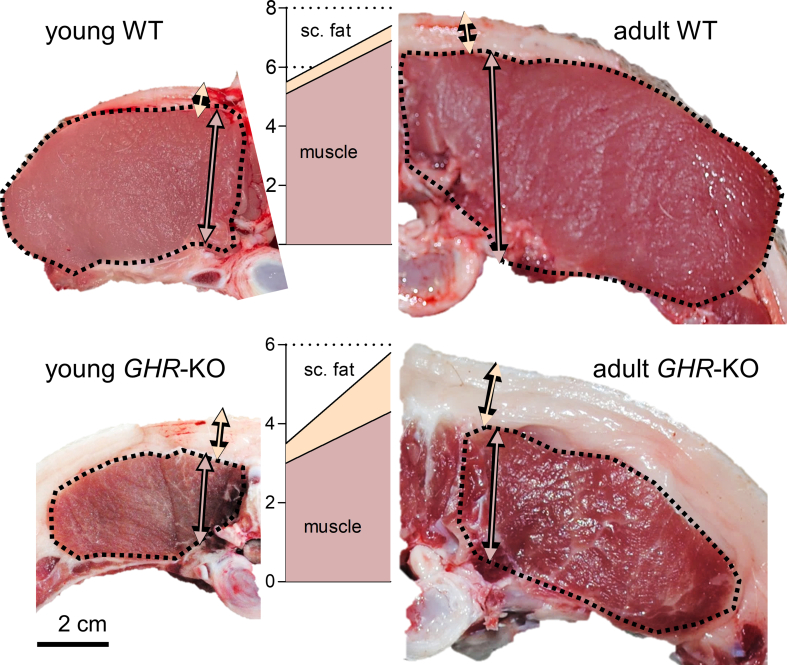
Representative cross-sections of subcutaneous adipose tissue/longissimus lumborum muscle at the level of the last rib in a young vs adult wild-type control (top) and young and adult *GHR*-KO pig (bottom).

*GHR*-KO pigs displayed a significantly altered body composition, as they progressively accumulated adipose rather than skeletal muscle tissue (see Fig. S2). The ratio of subcutaneous adipose tissue to longissimus lumborum muscle height was already 2.7 times higher in young and 4.9 times in adult *GHR*-KO pigs vs. age-matched WT controls (p < 0.0001, Figure 1D), while the body composition in WT pigs did not change with age.

### 3.2 Serum cortisol levels are higher in *GHR*-KO pigs

Serum cortisol levels were 96% and 21% higher in young and adult *GHR*-KO vs age-matched WT pigs (p = 0.0364; Table S3). Serum corticosterone and deoxycortisone levels were not affected by genotype nor age. Cortisone and aldosterone levels decreased with age (p = 0.0042, p = 0.1187) but were not affected by group (p = 0.9481, p = 0.1225).

### 3.3 Sex hormones had no effect on body composition or metabolism of adult *GHR*-KO pigs

**Figure S3:**
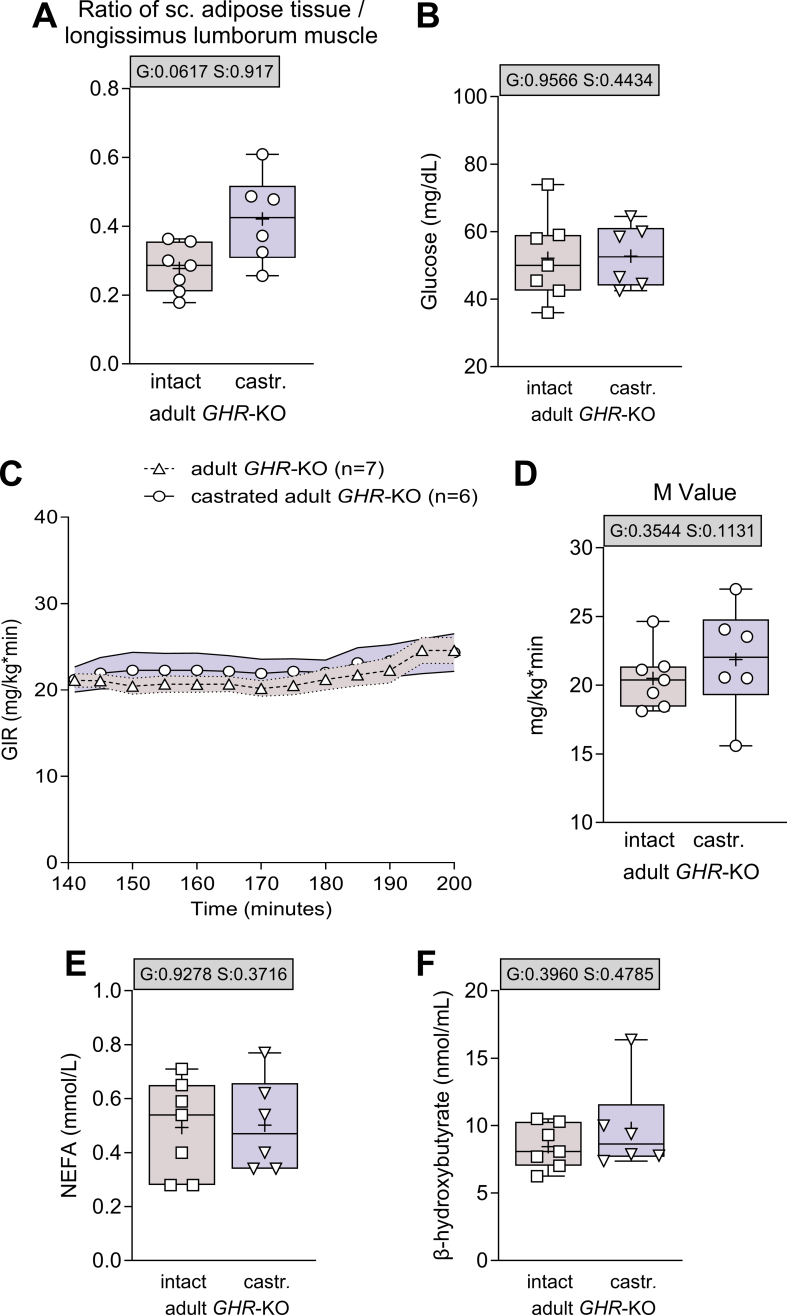
Effect of castration at infancy on adult *GHR*-KO pigs. (**A**) Fat-to-muscle ratio (thickness of overlaying subcutaneous adipose tissue / longissimus lumborum muscle height). (B) Glucose, (C) Glucose infusion rate (GIR) in intact vs. early castrated GHR-KO pigs at adult age, (D) M-value, (E) NEFA, (F) β-hydroxybutyrate levels. The box plots show median, interquartile range (box) and extremes (whiskers). The mean is marked as “+”. Results of analysis of variance are indicated: G: p-value of the effect of Group; S: p-value of the effect of Sex.

Early castration of *GHR*-KO pigs resulted in diminished sex hormones at adult age (Table S4) and in a mild, insignificant increase in subcutaneous adipose tissue to muscle ratio compared to intact *GHR*-KO pigs (p = 0.0617; Fig. S3A). Importantly, castration did not prevent normalization of glucose levels at adult age (Fig. S3B). Insulin sensitivity was similar in castrated and intact *GHR*-KO pigs (Fig. S3C and D). Solely serum triglyceride levels decreased after castration (14.6 ± 2.7 mg/dL vs. 24.5 ± 2.6 mg/dL in intact *GHR*-KO pigs; p = 0.0266), while other clinical–chemical parameters, such as NEFA or BHB (Fig. S3E and F), were similar in early castrated and intact *GHR*-KO pigs at adult age. Except for sexual hormones, none of the investigated parameters was significantly influenced by sex at adult age (the most relevant parameters are shown in Table S5). A notable increase in testosterone levels was observed in male adult *GHR*-KO compared to WT pigs (p < 0.0001; Table S5).

### 3.4 *GHR*-KO pigs show increased insulin sensitivity, particularly at young age

**Figure 2:**
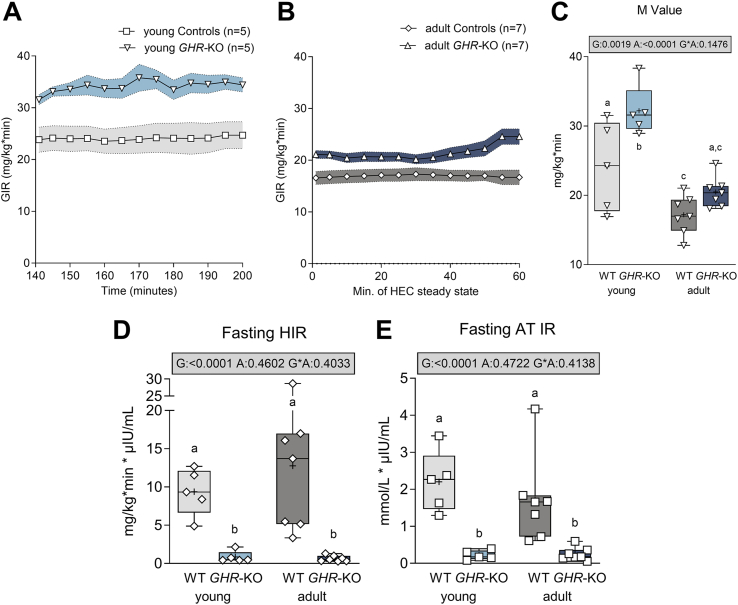
Hyperinsulinemic-euglycemic clamp (HEC) study of young and adult *GHR*-KO pigs and WT controls. (**A**, **B**) Glucose infusion rate (GIR) in young (**A**) and adult (**B**) pigs. M-value (**C**), fasting hepatic **(D)** and adipose tissue insulin resistance **(E)**. The box plots show median, interquartile range (box) and extremes (whiskers). The mean is marked as “+”. Results of analysis of variance are indicated: G: p-value of the effect of Group; A: p-value of the effect of Age; G∗A: p-value of the interaction Group∗Age. Means with different superscript letters are significantly different (p < 0.05).

Glucose infusion rate (GIR, Figure 2A,B) during HEC and whole-body insulin sensitivity (M-value, mainly representing skeletal muscle insulin sensitivity, Figure 2C, Table S6) were higher in young than in adult animals (WT: +40%; *GHR*-KO: +57 %; p < 0.0001 for the effect of age). *GHR*-KO pigs exhibited an increased M-value compared with WT pigs (p = 0.0019 for the effect of group). This was more pronounced in the juvenile (M value: 32.2 ± 1.6 in *GHR*-KO vs. 24.1 ± 2.9 mg/kg∗min in WT controls; +34%; p = 0.0025) than in the adult age group (M value: 20.5 ± 1.4 in *GHR*-KO vs. 17.2 ± 1.1 mg/kg∗min in WT controls; +20%; p = 0.1239).

Fasting HIR and AT IR were lower in *GHR*-KO pigs compared to WT controls (p < 0.0001 and p < 0.0001 for the effect of group; Figure 2D,E). NEFA suppression during HEC, representing AT insulin sensitivity, was unaltered in *GHR*-KO pigs and not affected by age (Table S6).

### 3.5 Reduced S660 phosphorylation of hormone sensitive lipase, especially in young *GHR*-KO pigs

**Figure 3:**
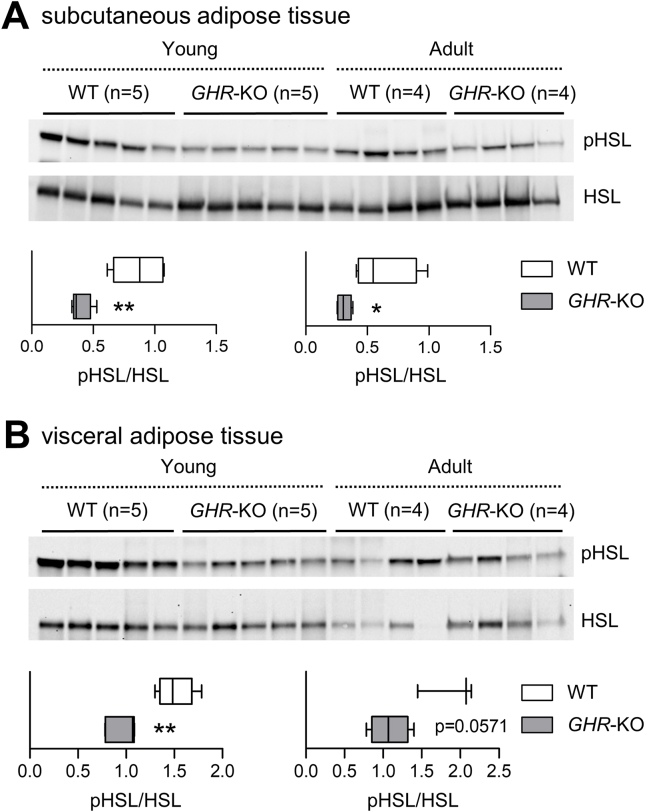
Western blot analysis of hormone sensitive lipase (HSL) S660 phosphorylation in subcutaneous (**A**) and visceral (**B**) adipose tissue. Significant differences in the ratio of phosphorylated to total HSL (pHSL/HSL) are indicated by asterisks. ∗p < 0.05; ∗∗p < 0.01.

As a proxy for hormone sensitive lipase (HSL) activity, we determined its S660 phosphorylation status [35] in both visceral and subcutaneous fat depots. Serine 660 phosphorylation of HSL correlates best with lipolysis activity as shown by mutagenesis experiments on rat adipocytes in vitro [36] as well as in human in vivo studies on lipolysis activity in response to stimulation [37]. HSL phosphorylation was decreased in both fat depots of young *GHR*-KO vs. WT pigs (p < 0.01), suggesting reduced activity. In adult *GHR*-KO pigs, HSL activity was significantly reduced in subcutaneous fat tissue (p < 0.05), while only borderline significance (p = 0.0571) was found for visceral fat (Figure 3).

### 3.6 Decreased EGP and depleted liver glycogen stores particularly in young *GHR*-KO pigs

**Figure 4:**
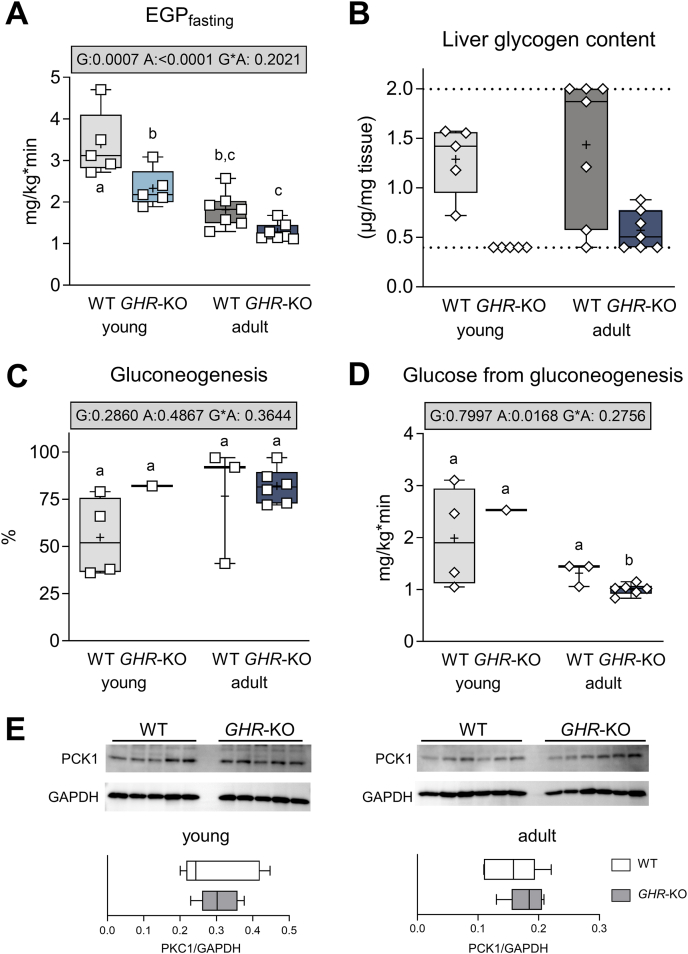
Endogenous glucose production in *GHR*-KO pigs and WT controls. (**A**) Fasting endogenous glucose production. (**B**) Liver glycogen content. (**C**) Contribution of gluconeogenesis to EGP. (**D**) glucose from gluconeogenesis determined by the ^2^H_2_O approach (numbers of animals assessed within the distinct group are indicated by the corresponding dots). (**E**) Western blot analysis of phosphoenolpyruvate carboxykinase 1 (PCK1) abundance in liver samples from young and adult WT and *GHR*-KO pigs. The box plots show median, interquartile range (box) and extremes (whiskers). The mean is marked as “+”. Results of analysis of variance are indicated: G: p-value of the effect of Group; A: p-value of the effect of Age; G∗A: p-value of the interaction Group∗Age. Means with different superscript letters are significantly different (p < 0.05).

Overall, EGP_fasting_ was lower in adult than in juvenile animals (p < 0.0001 for the effect of age) and in *GHR*-KO than in WT pigs (p = 0.0007 for the effect of group; Figure 4A, Table S6).

This was associated with a complete and partial reduction of liver glycogen stores in young and adult *GHR*-KO pigs, respectively (Figure 4B). In the juvenile age group, glycogen content averaged 1.29 ± 0.4 μg/mg in WT liver samples, but was consistently below the detection limit (0.4 μg/mg) in *GHR*-KO liver samples. In the adult group, liver glycogen was detectable in four of seven *GHR*-KO pigs (0.7 ± 0.2 μg/mg), whereas WT animals generally exhibited higher values, with three exceeding the upper detection limit (2.0 μg/mg) and others averaging 1.2 ± 0.6 μg/mg.

To determine whether reduced EGP was due to diminished glycogenolysis or gluconeogenesis, we applied the ^2^H_2_O tracer method in adult pigs. Sufficient body water enrichment of the ^2^H_2_O tracer was achieved in n = 3 adult WT, n = 5 adult *GHR*-KO, and n = 4 young WT pigs, but unfortunately only in n = 1 young *GHR*-KO pig. Analysis of variance did not reveal a significant overall effect for group (p = 0.2860; Figure 4C; Table S6). Within the adult age group, the proportion of gluconeogenesis did not differ significantly between genotypes (*GHR*-KO: 79 ± 9%; WT: 77 ± 18%; Figure 4C), nor did the absolute rate of gluconeogenesis-derived glucose (1.1 ± 0.2 vs. 1.3 ± 0.2 mg/kg∗min; Figure 4D). Similar glucagon levels (p = 0.1463; Table S2) and comparable hepatic expression of phosphoenolpyruvate carboxykinase 1 (PCK1; Figure 4E) across age and genotype further suggest that gluconeogenic capacity was not impaired.

### 3.7 Juvenile hypoglycemia in *GHR*-KO pigs is associated with increased BHB but not NEFA levels

**Figure 5:**
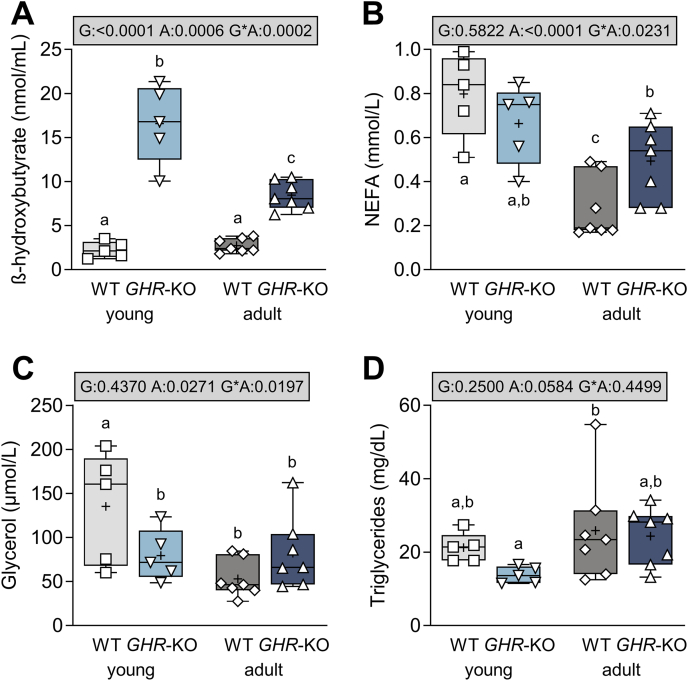
Fasting serum levels of (**A**) β-hydroxybutyrate, (**B**) non-esterified fatty acids (NEFA), (**C**) glycerol, and (**D**) triglycerides in young and adult *GHR*-KO pigs and WT controls. The box plots show median, interquartile range (box) and extremes (whiskers). The mean is marked as “+”. Results of analysis of variance are indicated: G: p-value of the effect of Group; A: p-value of the effect of Age; G∗A: p-value of the interaction Group∗Age. Means with different superscript letters are significantly different (p < 0.05).

At young age, fasting BHB levels of *GHR*-KO pigs were on average 7.2 times higher than in WT controls (16.6 ± 2.0 vs. 2.3 ± 0.4 nmol/mL; p < 0.0001; Figure 5A). At adult age, BHB levels of *GHR*-KO pigs were 3.1 times higher than in age-matched controls (8.5 ± 0.9 vs. 2.7 ± 0.3 nmol/mL; p = 0.0001), whose values remained relatively constant (p = 0.0002 for the interaction of group∗age).

Despite hypoglycemia and ketosis, young *GHR*-KO pigs showed no increase in NEFA levels, which were in fact slightly decreased in comparison with young controls (0.70 ± 0.07 vs. 0.80 ± 0.08 mmol/L; p = 0.2174; Figure 5B, Table S2). NEFA levels were generally higher in young animals (p < 0.0001 for the effect of age), with a pronounced age-dependent decrease was observed mainly in WT pigs (p = 0.0231 for the interaction of group∗age). In adult *GHR*-KO pigs, displaying normoglycemia and less pronounced ketosis, NEFA levels were higher than in adult WT controls (0.50 ± 0.06 vs. 0.30 ± 0.06 mmol/L; p = 0.0299). Similar trends were observed for glycerol with lower levels in young *GHR*-KO compared to WT pigs (79.6 ± 13.1 vs. 135.2 ± 28.5 μmol/L; p = 0.0290) and no age-related decrease in glycerol levels as observed in WT pigs (p = 0.0197 for the interaction of group∗age; Figure 5C, Table S2).

Fasting triglyceride levels were as a trend decreased by 35% in young and 24% in adult *GHR*-KO pigs (p = 0.2500 for the effect of group; Figure 5D; Table S2). Serum levels of cholesterol as well as HDL and VDL cholesterol were similar in *GHR*-KO and WT pigs and displayed an overall decrease with age (p = 0.0105 and p = 0.0554 for the effect of age; Table S2).

### 3.8 Increased fatty acid beta-oxidation and utilization of glucogenic amino acids in *GHR*-KO pigs

**Figure S4:**
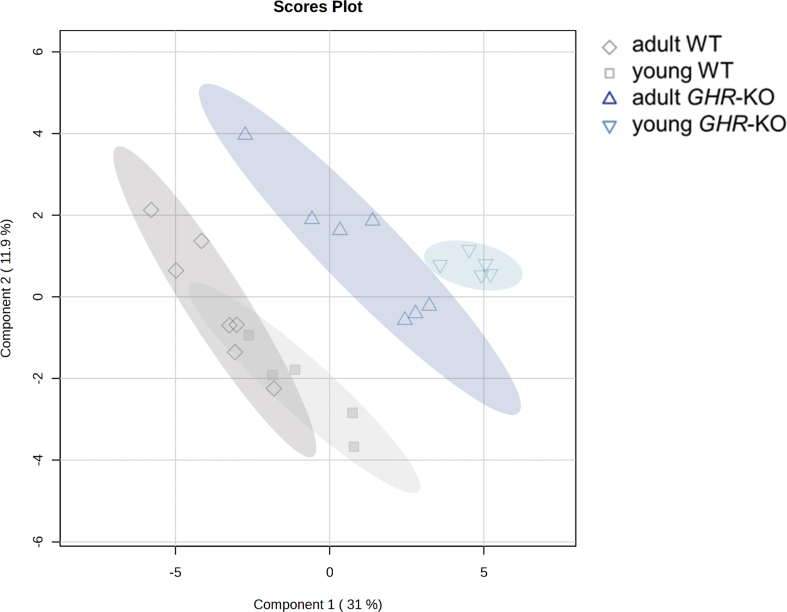
Partial Least Squares Discriminant Analysis (PLS-DA) of targeted metabolomic profiles in young and adult *GHR*-KO and WT pigs.

To elucidate metabolic alterations in *GHR*-KO pigs in greater detail, we analyzed serum samples by targeted metabolomics. Specifically, we aimed to understand how elevated BHB levels can be maintained without a corresponding increase in NEFA concentrations, particularly in young *GHR*-KO pigs. In total, 187 metabolites, including 39 acylcarnitines, free carnitine, 21 amino acids, 21 biogenic amines, 14 lysophosphatidylcholines, 76 phosphatidylcholines, and 15 sphingolipids, were quantified and physiologically relevant ratios calculated (Tables S7–12). Partial Least Squares Discriminant Analysis (PLS-DA) clearly separated metabolomic profiles from *GHR*-KO and control samples (Fig. S4).

**Figure 6:**
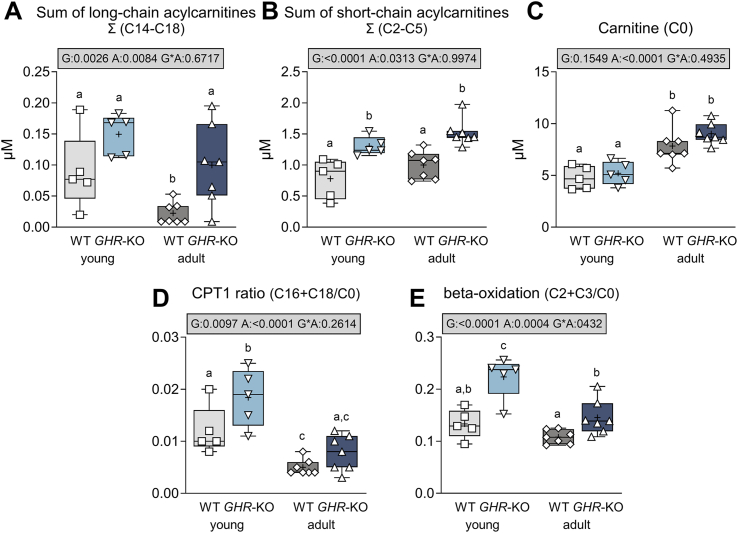
Acylcarnitine levels in *GHR*-KO pigs and WT controls. (**A**) Sum of long-chain acylcarnitines. (**B**) Sum of short-chain acylcarnitines. (**C**) Free carnitine. (**D**) CPT1 ratio as a proxy for the activity of carnitine palmitoyltransferase 1A (CPT1A). (**E**) Ratio of acetylcarnitine (C2) and propionylcarnitine (C3) to free carnitine (C0) as a measure for beta-oxidation activity. The box plots show medians, interquartile range (box) and extremes (whiskers). The mean is marked as “+”. Results of analysis of variance are indicated: G: p-value of the effect of Group; A: p-value of the effect of Age; G∗A: p-value of the interaction Group∗Age. Means with different superscript letters are significantly different (p < 0.05).

Our results indicate an increased beta–oxidative activity in *GHR*-KO pigs, as increased concentrations of long-chain (C14–C18), as well as short-chain acylcarnitines (C2–C5) were detected (p = 0.0026 and p < 0.0001 for the effect of group; Figure 6A,B), while carnitine (C0; Figure 6C) levels were unaltered. An increased mitochondrial uptake of NEFAs via the carnitine palmitoyltransferase 1A (CPT1A) in *GHR*-KO pigs is indicated by an increased ratio of long-chain acylcarnitines (C16 + C18) to free carnitine (C0) (CPT1 ratio; p = 0.0097 for the effect of group; Figure 6D). Overall, the CPT1A activity was higher in young than in adult animals (p < 0.0001 for the effect of age). An increased ratio of C2 + C3 acylcarnitines to C0 in *GHR*-KO pigs (p < 0.0001 for the effect of group; Figure 6E) further indicates an increased beta-oxidation rate [38], degrading NEFAs to acetyl-CoA as a substrate for ketogenesis and explaining the highly elevated BHB levels in young *GHR*-KO pigs.

**Figure 7:**
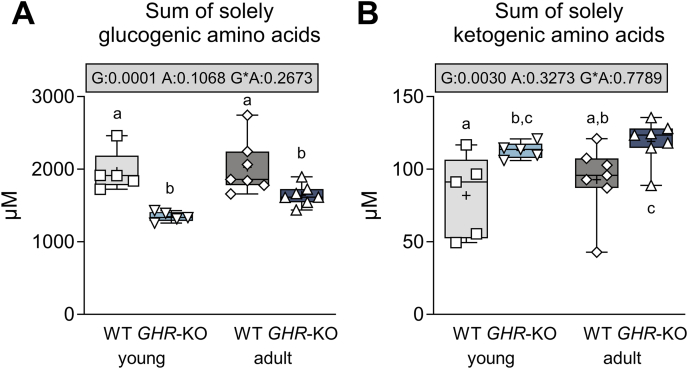
Levels of solely glucogenic (**A**) and ketogenic amino acids (**B**) in *GHR*-KO pigs and WT controls. The box plots show medians, interquartile range (box) and extremes (whiskers). The mean is marked as “+”. Results of analysis of variance are indicated: G: p-value of the effect of Group; A: p-value of the effect of Age; G∗A: p-value of the interaction Group∗Age. Means with different superscript letters are significantly different (p < 0.05).

An increased utilization of amino acids as substrates for gluconeogenesis in *GHR*-KO pigs is indicated by significantly decreased plasma concentrations of solely glucogenic amino acid (p = 0.0001 for the effect of group, Figure 7A) while levels of solely ketogenic amino acids were increased (p = 0.003 for the effect of group, Figure 7B). Notably, we observed decreased levels of branched chain amino acids (BCAAs, p = 0.0344; Table S8) in *GHR*-KO pigs, which is of particular interest, as increased BCAA levels are considered as risk factor for the development of diabetes [39].

**Figure S5:**
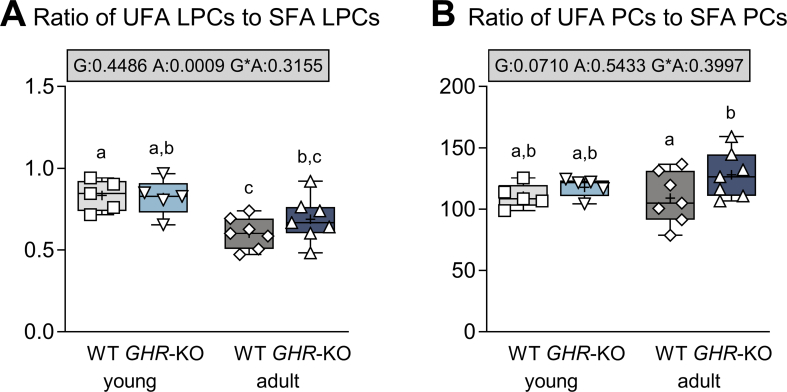
Ratio of unsaturated (UFA) to saturated (SFA) lysophosphatidylcholines (LPCs, **A**) phosphatidylcholines (PCs, **B**) in *GHR*-KO pigs and WT controls. The box plots show median, interquartile range (box) and extremes (whiskers). The mean is marked as “+”. Results of analysis of variance are indicated: G: p-value of the effect of Group; A: p-value of the effect of Age; G∗A: p-value of the interaction Group∗Age. Means with different superscript letters are significantly different (p < 0.05).

The lipid profile of *GHR*-KO pigs was largely comparable to that of WT controls, as the ratio of unsaturated to saturated lysophosphatidylcholines showed no significant group-related differences (Fig. S5A), and the corresponding ratio for phosphatidylcholines was only mildly elevated in *GHR*-KO pigs (p = 0.0710 for the effect of group; Fig. S5B).

### 3.9 Adipose tissue accumulation in *GHR*-KO pigs without major canonical gene expression alterations

**Figure S6:**
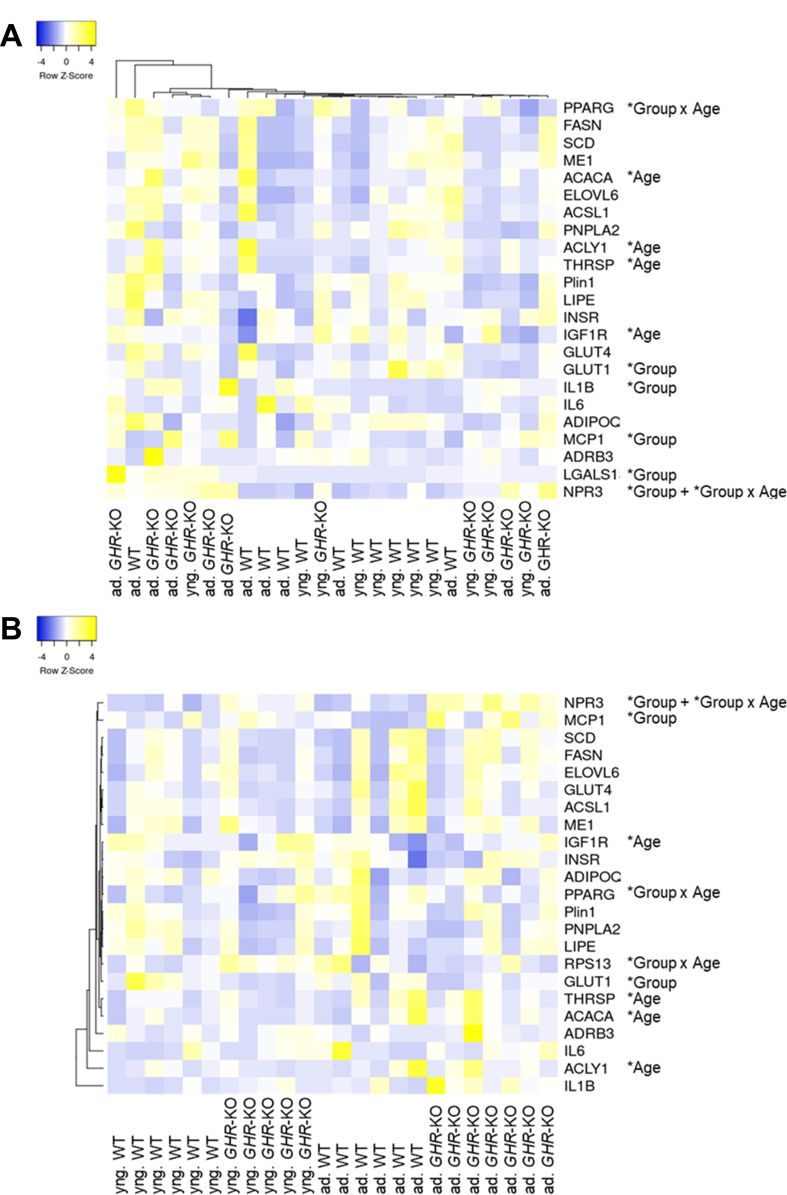
Heat map of the expression pattern of genes involved in lipolysis, adipogenesis and lipid desaturation in *GHR*-KO vs. WT subcutaneous adipose tissue, clustered for genes (**A**) and groups (**B**). Effects of analysis of variance are indicated when showing p-value <0.05 for Group, Age and/or Group x Age.

Since a previous study in *GHR*-KO pigs found many gene sets and pathways related to metabolism enriched in the transcriptome of predominantly subcutaneous but not visceral adipose tissue [15], we evaluated the expression pattern of selected genes involved in adipogenesis, lipolysis as well as glucose metabolism, insulin signaling, and inflammation via qPCR in the subcutaneous depot. Remarkably, the expression pattern appeared less different than expected from the obvious degree of obesity in *GHR*-KO pigs (see Fig. S6, Table S13). The transcript levels of genes involved in lipid and fatty acid synthesis such as *PPARG*, *FASN*, *SCD*, *ME1*, *ACACA* and *EVOVL6* were not significantly altered in *GHR*-KO adipose tissue. The unaltered mRNA expression for stearoyl-CoA desaturase (SCD; p = 0.9275), an enzyme that introduces a double bond into saturated fatty acids, converting them into monounsaturated fatty acids [40], aligns with lipid profiles assessed for *GHR*-KO pigs. Higher *NPR3* transcript levels in *GHR*-KO pigs (p < 0.0001) are interesting since the corresponding protein can block some of the lipolytic action usually induced by natriuretic peptides [41]. Obesity-related desensitization towards insulin action in adipose tissue is commonly associated with a reduced expression of the *INSR* [42], which was not the case in *GHR*-KO subcutaneous adipose tissue. Further, the expression of *GLUT4*, which represents a marker for systemic insulin sensitivity [42], appeared unaltered. While an increased expression of *MCP1* (p = 0.006) can indicate low-grade inflammation in association with obesity [43], *IL6* and *IL1B* transcript levels were not or only as a trend increased, which appears in line with a protection against obesity-related adipose tissue inflammation observed in *Ghr*-KO mice [44]. Taken together, our results indicate that the increased accumulation of adipose tissue in *GHR*-KO pigs does not lead to the common obesity-related pathophysiology.

## 4 Discussion

*GHR*-KO pigs reflect the phenotype of increased insulin sensitivity despite obesity observed in human LS patients [5]. While the fasting period was similar in all animals, young *GHR*-KO pigs displayed a pronounced negative energy balance potentially due to reduced substrate availability. That includes depleted liver glycogen stores, contributing to reduced hepatic glucose output, juvenile hypoglycemia and increased ketogenesis. Our data further indicates an increased utilization of NEFA for beta-oxidation combined with a decreased NEFA release from adipose tissue lipolysis, both contributing to a lack of increase in circulating NEFA despite elevated BHB levels.

Our results of decreased fasting EGP and reduced fasting HIR and AT IR mirror the opposite effects of GH on glucose metabolism assessed in acromegaly. In those patients, GH excess results in a lean but insulin-resistant phenotype (reviewed in [45]). Arlien-Søborg et al. [46] investigated metabolic properties of patients with acromegaly using [3-^3^H]-glucose tracers in combination with hyperinsulinemic-euglycemic clamp studies before and after disease control. Within that study, it was assessed that GH overabundance is associated with increased EGP [46,47]. Also, increased AT IR was present in patients with acromegaly [46] due to the function of GH-mediated NEFA release from adipose tissues (reviewed in [48]).

Our study identified increased insulin sensitivity as the major driver of juvenile hypoglycemia in *GHR*-KO pigs. Hypoglycemia due to increased insulin sensitivity is also reported for human type 2 diabetic patients after treatment with insulin-sensitizing drugs [49,50] and after gastric bypass surgery [51]. Under physiological conditions, GH provides major counter-regulatory responses to hypoglycemia, stimulating lipolysis and inducing insulin resistance (reviewed in [2,45,52]). In its absence, GH-insensitive individuals face impaired glucose homeostasis, with fasting hypoglycemia - even below 30 mg/dL - reported in children with Laron syndrome [53]. Resembling that phenotype, young hypoglycemic *GHR*-KO pigs displayed markedly reduced fasting endogenous glucose production and diminished lipolytic activity, resulting in limited NEFA availability. To compensate, these animals shifted toward enhanced ketogenesis, fueled by increased beta-oxidation and acetyl-CoA production, further depleting NEFA reserves. Previous investigations of functional changes in the liver using proteomics and metabolomics revealed alterations in fatty acid and amino acid metabolism related pathways in 6-month-old *GHR*-KO pigs [54]. This study inter alia detected 3-hydroxy-3-methylglutaryl-CoA synthase 2 as the protein with the highest abundance increase in *GHR*-KO liver samples. As the key enzyme for ketogenesis, it converts acetyl-CoA and acetoacetyl-CoA into the key intermediate for the synthesis of ketone bodies (reviewed in [55]). The utilization of amino acids as gluconeogenic substrates in *GHR*-KO pigs corresponds with generally increased levels of proteins especially involved in amino acid catabolism and the TCA cycle in the liver [54] and can contribute to the phenotype of reduced muscle mass in GH insensitivity [11]. Children generally have a higher rate of ketogenesis during fasting [56], implying the functional importance of GH-mediated lipolysis in early life. This is supported by the higher NEFA levels in young compared to adult WT pigs. This physiological higher availability of NEFA at young age is lacking in *GHR*-KO pigs as well as in patients with GH insensitivity or deficiency, possibly contributing to their metabolic problems at young age.

In line with the phenotype of human patients, we observed a normalization of fasting glucose levels in *GHR*-KO pigs at adult age. To explain this phenomenon in GH-insensitive or deficient patients, several hypotheses have been proposed, including a decrease in insulin sensitivity [53] due to the impact of rising sex hormone concentrations during puberty [7] and an involvement of the increasing accumulation of adipose tissues [57]. In fact, we observed a decrease in insulin sensitivity in adult *GHR*-KO pigs, compared to their young counterparts. Nevertheless, insulin sensitivity was still increased in comparison with age-matched WT controls. The decrease in insulin sensitivity was accompanied by a decrease in fasting insulin levels in adult *GHR*-KO pigs. A more pronounced decrease in insulin secretion at adult age has been shown in previous studies in young and adult *GHR*-KO pigs [16]. A decrease in insulin secretion can serve as a compensatory mechanism on insulin hypersensitivity [58] and lead to normoglycemia at adult age. Conversely, the insulin hypersensitivity in young *GHR*-KO pigs may also be a consequence of their reduced insulin secretory capacity. A counterregulatory increase in insulin sensitivity is described upon the loss of insulin secretory capacity in hereditary haemochromatosis [59]. The metabolic state in adult *GHR*-KO pigs can be characterized as less catabolic than in young animals, including the maintenance of normoglycemia and a less pronounced ketosis. The dampened insulin sensitivity in adult *GHR*-KO pigs allowed a sufficient release of NEFAs from adipose tissues, which accumulated with age.

Puberty is commonly associated with a decrease in insulin sensitivity and a compensatory increase in insulin secretion (reviewed in [9]). This is indeed resembled in WT pigs, in line with previous studies [16]. In some LS patients, it has been observed that the transition to normoglycemia occurred around the age when sexual maturity was attained, but this was not recapitulated in *Ghr*-KO mice (reviewed in [9]). Our present study shows that sex hormones are not causal for the normalization of blood glucose levels in GH insensitivity as early castration of *GHR*-KO pigs did not preserve the hypoglycemic phenotype into adulthood.

The lack of lipolytic GH action drives the accumulation of adipose tissue in GH insensitivity (reviewed in [48]). In *Ghr*-KO mice, a preferential accumulation of subcutaneous adipose tissue is commonly seen in association with improved insulin sensitivity [60] and *Ghr*-KO mice remain insulin sensitive when fed a high-fat diet (reviewed in [4]). Further, adipose tissue from *Ghr*-KO mice even improves insulin sensitivity when transplanted into wild-type mice [61]. A direct comparison of adipose tissue morphology and transcriptome revealed similarities between human LS patients and *GHR*-KO pigs regarding adipocyte size and gene expression profile [15]. Our results show that the increase in adipose tissue in GH insensitivity is not strictly associated with the common obesity-related pathophysiology as a mechanism to promote insulin resistance with age. On the other hand, we observed a decreased HSL activity mainly in young *GHR*-KO pigs, while the trend towards normalization of HSL activity in adult animals was associated with a more sufficient NEFA release upon fasting and dampened insulin sensitivity. It has to be noted, that the activity of HSL has solely been assessed within the distinct age groups while a direct *in vivo* or *ex vivo* estimation of lipolytic activity could help to assess the age-specific activity.

These observations prompt the fundamental question, by what mechanisms the enhanced insulin sensitivity in GH insensitivity is mediated. Previous studies in *GHR*-KO pigs revealed a decreased pancreatic beta-cell volume, which was associated with a decreased insulin secretory capacity [16]. In spite of the generally low insulin levels in GH insensitivity, *GHR*-KO pigs displayed a preserved glucose tolerance as observed in human LS patients [5], most likely due to the markedly increased insulin sensitivity. This effect is likely attributable to both, a direct modulation of insulin signaling pathways, and indirect mechanisms associated with broader metabolic alterations. Evidence from *Ghr*-KO mice indicates an increased expression of hepatic insulin receptors, along with augmented phosphorylation of downstream signaling components [62]. That is seen as a compensatory mechanism to the decreased insulin secretory capacity [16], contributing to an elevated responsiveness to insulin stimulation in the absence of GH signaling (reviewed in [2]). In *GHR* intact individuals, GH promotes lipolysis and the release of NEFAs, which directly impair insulin sensitivity. The accumulation of lipids in insulin-responsive tissues is considered a key driver of lipid-induced insulin resistance (reviewed in [63]). In particular, the intramyocellular buildup of diacylglycerol (DAG) and ceramides, NEFA-derived signaling intermediates, has been shown to impair insulin signaling and reduce glucose uptake in skeletal muscle (reviewed in [64,65]). Further, it has been discussed, that NEFA oxidation suppresses pyruvate dehydrogenase activity, raising intracellular glucose-6-phosphate concentrations, which prevents glucose uptake, contributing to fat-induced insulin resistance by inhibiting insulin-stimulated glucose uptake ([66,67], reviewed in [64]). In line with that, the transition towards normoglycemia with age appears directly linked to body adipose tissue content in hypopituitary children, and lean GH-deficient children are more prone to show symptomatic hypoglycemia ([57], reviewed in [68]). From this perspective, we propose a dynamic model in which an increased release of NEFAs from accumulating adipose tissue with age triggers a reduction of the exaggerated insulin sensitivity. This may establish a self-reinforcing cycle, decreasing the insulin-mediated suppression of HSL activity and NEFA release observed in adult *GHR*-KO pigs. Future studies of arteriovenous metabolomics [69] in *GHR*-KO pigs can directly assess the remodeling of metabolic flux between organs and tissues. This approach can determine to what extent the lack of a corresponding increase in NEFA levels during increased ketogenesis in young *GHR*-KO pigs is due to increased utilization of free fatty acids or to insufficient release from adipose tissues.

## 5 Conclusion

This study uncovers insulin sensitivity–driven ketotic hypoglycemia as a core consequence of GH insensitivity in young *GHR*-KO pigs, linked to impaired lipolysis and altered substrate utilization during development. The age-dependent restoration of metabolic balance appears to be associated with progressive fat mass accumulation, but not with rising sex hormones. An adequate NEFA release in adult *GHR*-KO pigs can contribute to a relative decline in insulin sensitivity compared to their younger counterparts. Reduced insulin secretion is another counterregulatory mechanism to avoid hypoglycemia in adult *GHR*-KO pigs.

## CRediT authorship contribution statement

**Arne Hinrichs:** Writing – original draft, Investigation, Formal analysis, Data curation, Conceptualization. **Kalliopi Pafili:** Writing – review & editing, Methodology, Formal analysis. **Gencer Sancar:** Writing – review & editing, Resources, Methodology, Investigation. **Laeticia Laane:** Investigation. **Silja Zettler:** Investigation. **Malek Torgeman:** Data curation. **Barbara Kessler:** Investigation. **Judith Leonie Nono:** Methodology. **Sonja Kunz:** Methodology. **Birgit Rathkolb:** Methodology. **Cristina Barosa:** Methodology. **Cornelia Prehn:** Methodology. **Alexander Cecil:** Data curation. **Simone Renner:** Methodology, Investigation, Conceptualization. **Elisabeth Kemter:** Methodology. **Sabine Kahl:** Methodology. **Julia Szendroedi:** Methodology, Conceptualization. **Martin Bidlingmaier:** Methodology. **John Griffith Jones:** Methodology. **Martin Hrabĕ de Angelis:** Methodology. **Michael Roden:** Writing – review & editing, Supervision, Methodology. **Eckhard Wolf:** Writing – review & editing, Supervision, Methodology.

## Funding

This study was funded by the Deutsche Forschungsgemeinschaft (DFG, German Research Foundation; HI 2206/2-1; FOR 5795; Project number 441815498; 536691227; CRC-TR 127; CRC-TR 205), by the German Center for Diabetes Research (DZD; FKZ 82DZD08D03) and the Portuguese Foundation for Science and Technology (FCT-FEDER-02/SAICT/2017/028147 and UIDB/Multi/04462/2020). Structural funding for the Center for Neurosciences and Cell Biology and the UC-NMR facility is supported in part by FEDER – European Regional Development Fund through the COMPETE Programme, Centro 2020 Regional Operational Programme, and the Portuguese Foundation for Science and Technology through grants UIDB/04539/2020; UIDP/04539/2020, LA/P/0058/2020 POCI-01-0145-FEDER-007440; REEQ/481/QUI/2006, RECI/QEQ-QFI/0168/2012, CENTRO-07-CT62-FEDER-002012, and Rede Nacional de Ressonancia Magnética Nuclear. The research of KP is supported by the Deutsche Forschungsgemeinschaft (DFG, German Research Foundation): Project number 493659010 in the context of the Clinician Scientist Program (FUTURE-4-CSPMM) and by grants from the German Diabetes Research (DZD) foundation and the Heinrich-Heine-University Düsseldorf.

## Declaration of competing interest

The authors declare that they have no known competing financial interests or personal relationships that could have appeared to influence the work reported in this paper.

## Data Availability

Data will be made available on request.
