# Corrigendum to “Transient juvenile hypoglycemia in GH insensitive Laron syndrome pigs is associated with insulin hypersensitivity” [Mol Metabol (2025) 102273]

**DOI:** 10.1016/j.molmet.2025.102286

**Published:** 2025-11-27

**Authors:** Arne Hinrichs, Kalliopi Pafili, Gencer Sancar, Laeticia Laane, Silja Zettler, Malek Torgeman, Barbara Kessler, Judith Leonie Nono, Sonja Kunz, Birgit Rathkolb, Cristina Barosa, Cornelia Prehn, Alexander Cecil, Simone Renner, Elisabeth Kemter, Sabine Kahl, Julia Szendroedi, Martin Bidlingmaier, John Griffith Jones, Martin Hrabĕ de Angelis, Michael Roden, Eckhard Wolf

**Affiliations:** 1 Chair for Molecular Animal Breeding and Biotechnology, Gene Center and Department of Veterinary Sciences, LMU Munich, Munich, Germany; 2 Center for Innovative Medical Models (CiMM), LMU Munich, Oberschleissheim, Germany; 3 Department of Endocrinology and Diabetology, Medical Faculty and University Hospital Düsseldorf, Heinrich-Heine-University Düsseldorf, Düsseldorf, Germany; 4 Institute for Clinical Diabetology, German Diabetes Center, Leibniz Center for Diabetes Research at Heinrich-Heine-University Düsseldorf, Düsseldorf, Germany; 5 German Center for Diabetes Research (DZD), Neuherberg, Germany; 6 Institute for Diabetes Research and Metabolic Diseases of the Helmholtz Center Munich, Tübingen, Germany; 7 Department of Internal Medicine IV, Division of Diabetology, Endocrinology and Nephrology, University Hospital of Tübingen, Tübingen, Germany; 8 Endocrine Laboratory, Medizinische Klinik und Poliklinik IV, Klinikum der Universität München, Munich, Germany; 9 Institute of Experimental Genetics, German Mouse Clinic (GMC), Helmholtz Center Munich, Neuherberg, Germany; 10 Center for Neurosciences and Cell Biology, UC Biotech, Cantanhede, Portugal; 11 Metabolomics and Proteomics Core, Helmholtz Center Munich, Neuherberg, Germany; 12 Department of Endocrinology, Diabetology, Metabolism and Clinical Chemistry, Heidelberg University Hospital, Heidelberg, Germany; 13 Joint Heidelberg-IDC translational Diabetes Program, Helmholtz Center Munich, Neuherberg, Germany; 14 Portuguese Diabetes Association, Lisbon, Portugal; 15 Chair of Experimental Genetics, School of Life Science Weihenstephan, Technical University Munich, Freising, Germany; 16 Interfaculty Center for Endocrine and Cardiovascular Disease Network Modelling and Clinical Transfer (ICONLMU), LMU Munich, Munich, Germany

The authors regret <Not to name Yanislava Karusheva as a Co-Author of the Manuscript. Her Affiliations are: 3: Department of Endocrinology and Diabetology, Medical Faculty and University Hospital Düsseldorf, Heinrich-Heine- University Düsseldorf, Düsseldorf, Germany; 4: Institute for Clinical Diabetology, German Diabetes Center, Leibniz Center for Diabetes Research at Heinrich-Heine-University Düsseldorf, Düsseldorf, Germany; 5: German Center for Diabetes Research (DZD), Neuherberg, Germany. Her ORCID ID is 0000-0002-4000-7894. Her Authorship Contribution is Methodology and Investigation>.The authors would like to apologise for any inconvenience caused.]

The authors would like to apologise for any inconvenience caused.

