# Supplementary material for: Transient juvenile hypoglycemia in GH insensitive Laron syndrome pigs is associated with insulin hypersensitivity
Source: Mol Metab. 2025 Oct 20;103:102273. doi: 10.1016/j.molmet.2025.102273 (PMC12639633; doi:10.1016/j.molmet.2025.102273)
Supplement: Multimedia component 1 [file mmc1.docx]

**Supplementary Material 1.** Western blot analysis of signaling molecules in liver and adipose tissue samples.

Liver tissue samples were homogenized in Laemmli extraction buffer, and the protein content was determined by the bicinchoninic acid protein assay. 20 µg of total protein was separated by SDS-PAGE and transferred to PVDF membranes (Millipore) by electro-blotting. Membranes were washed in TBS with 0.1% Tween-20 and blocked in 5% w/v fat free milk powder (Roth) for 1 h. The membranes were then washed again and incubated in 5% w/v BSA (Roth) solution with the appropriate primary antibodies overnight at 4 °C.

Adipose tissue samples were lysed in cold lysis buffer (50 mM Tris, pH 7.5, 150 mM NaCl, 1 % NP40, 0.5 % NaDoc, 0.1 % SDS, 5 % glycerol, 1 mM EDTA, protease and phosphatase inhibitors (cOmplete and PhosSTOP (Roche)) by homogenization with bead-beater for 30 sec. After an initial centrifugation for 10 min at 18,000 × *g* at 4 °C, the middle clear phase was transferred to new tubes. A second 30-min centrifugation was performed, and the middle clear phase was transferred to new tubes. 5 μl protein extract was used for BCA assay to determine protein concentration. Samples were boiled in Laemmli buffer for 5 min at 95 °C. SDS-PAGE and blotting were performed using Trans-Blot Turbo Transfer System (Bio-Rad).

Phosphoenolpyruvate carboxykinase 1 (PCK1) abundance in liver samples was assessed using commercially available antibodies from Cell Signaling (#12940, 1:1000 for PCK1 and #2118, 1:4400 for GAPDH). S660 phosphorylation of hormone sensitive lipase (HSL) was assessed in adipose tissues using commercially available antibodies from Cell Signaling (#4107, 1:1000 for HSL and #45804, 1:1000 for pHSL-660).

After washing, the membranes were incubated in 5% w/v fat-free milk powder solution with the secondary HRP-linked goat anti-rabbit IgG (Bio-Rad 1706515, 1:5000 for adipose tissue samples; Cell Signaling #7074, 1:2000 for liver tissue samples) or HRP-goat anti-mouse IgG (Jackson ImmunoResearch,115-035-146, 1:5000) for 1 h. Bound antibodies were detected using the SuperSignal™ ECL reagent (Thermo Fisher Scientific) and appropriate films from the same supplier.
