# Supplementary material for: Transient juvenile hypoglycemia in GH insensitive Laron syndrome pigs is associated with insulin hypersensitivity
Source: Mol Metab. 2025 Oct 20;103:102273. doi: 10.1016/j.molmet.2025.102273 (PMC12639633; doi:10.1016/j.molmet.2025.102273)
Supplement: Multimedia component 2 [file mmc2.docx]

Gene Primer Sequence

*PPARG* forward GGAGATCACAGAGTATGCCAAG

reverse GAGGACCCCGTCTTTATTCATC

*FASN*  forward ATGGGCAAGCTGAAGGAC

reverse AAGGGAAGCAGGGTTGATG

*SCD* forward TGACCTAAAAGCCGAGAAGC

reverse TTCACCCCAGCAATACCAG

*ME1* forward CAGTTTAGCATTTCGGAAGCC

reverse AATACGTTCTCCATCAGTCACC

*ACACA* forward ACAGTGGAGCGAGAATTGG

reverse AATGGACAGAGTTGAGAGCAC

*ELOVL6* forward CATTAGTGCTGTGGTCTCTGAG

reverse GAATTTGCTGACAGGTCCATTG

*ACSL1*  forward TGATTCACAAGGGCTTCAAGG

reverse TTCAATGATCACCCACTCAGG

*PNPLA2* forward TTCATTCCCGTGTACTGCG

reverse TGATGGTGCTCTTGAGTTCG

*ACLY1*  forward CGATAGACAACCCAGACATGAG

reverse AACCGTCTACATTCAGGATAAGG

*THRSP* forward CAGAGACCGAAGAGACCAAG

reverse AGGGTAAGATGGGTAAGAATGTG

*PLIN1* forward CCTGAAAAGATCGCCTCTGAG

reverse ACGCTGATGCTGTTCCTG

*LIPE* forward GACGAGATTAGCACCAAGGAG

reverse ATTCTTGACGATGGGAGCTG

*INSR* forward CCCTCCGCCTCCTTCTTAT

reverse CTCCTGAGGCTATGGGACAG

*IGF1R* forward AGTACCGCAAGGTCTTCGAG

reverse GTTCAGGTCTGGGCACAAAG

*GLUT4* forward CCCAGAAGGTGATTGAACAGAG

reverse AAGGAAGAGAACATGCCACC

*GLUT1* forward TTCTCTGTGGGCCTGTTTGT

reverse CCATGAGCACAGCAGATATGA

*IL1B* forward GGCAGATGGTGTCTGTCATC

reverse CCTGGGAGGAGGGATTCTT

*IL6* forward AGGCAAAAGGGAAAGAATCC

reverse TCCACTCGTTCTGTGACTGC

*ADIPOQ* forward TGTCCCTAACATGCCCATTC

reverse CGTGATGTGGAAGGAGAAGTAG

*MCP1* forward TGTGCCTGCTGCTCACTG

reverse GCAGCAGGTGACTGGAGAAT

*ADRB3* forward GTGTCGTTCGCTCCTATCATG

reverse AAGGTAGAAGGAGACGGAGG

*NPR3* forward GAACATTTGAAGGTATTGCCGG

reverse CAATCACAGAGAAGTCCCCATAC

**Table S1.** Primers used for RT-qPCR analysis of subcutaneous adipose tissue.

PPARG, Peroxisome proliferator-activated receptor gamma; FASN. Fatty acid synthase; SCD, Stearoyl-CoA desaturase; ME1, NADP-dependent malic enzyme; ACACA, Acetyl-CoA carboxylase 1; ELOVL6, Very long chain fatty acid elongase 6; ACSL1, Long-chain-fatty-acid--CoA ligase 1; PNPLA2, Patatin-like phospholipase domain-containing protein 2; ACLY, ATP-citrate synthase; THRSP, Thyroid hormone-inducible hepatic protein; PLIN1, Perilipin-1; LIPE, Hormone-sensitive lipase; INSR, Insulin receptor; IGF1R, Insulin-like growth factor 1 receptor; GLUT4, Solute carrier family 2, facilitated glucose transporter member 4; GLUT1, Solute carrier family 2, facilitated glucose transporter member 1; IL1B, Interleukin-1 beta; IL6, Interleukin-6; ADIPOQ, Adiponectin; MCP1, Monocyte chemoattractant protein-1; ADRB3, Beta-3 adrenergic receptor;, NPR3, Natriuretic Peptide Receptor-3
