# Supplementary material for: Transient juvenile hypoglycemia in GH insensitive Laron syndrome pigs is associated with insulin hypersensitivity
Source: Mol Metab. 2025 Oct 20;103:102273. doi: 10.1016/j.molmet.2025.102273 (PMC12639633; doi:10.1016/j.molmet.2025.102273)
Supplement: Multimedia component 3 [file mmc3.docx]

Parameter young WT young *GHR*-KO adult WT adult *GHR*-KO Group Age Group*Age

Glucose (mg/dL) 74.5±3.9 39.5±3.9 58.1±3.3 52.1±3.3 **<0.0001** 0.6152 **0.0006**

Insulin (µIU/mL) 2.8±1.1 0.3±1.1 7.4±1.0 0.5±1.0 **0.0005 0.0444** 0.0620

C-Peptide (pmol/L) 41.1±11.0 19.0±11.0 82.8±9.3 19.0±10.7 **0.0015** 0.0897 0.0897

Glucagon (pmol/L) 1.7±0.2 1.6±0.1 2.2±0.3 6.1±1.1 0.1463 0.0556 0.1244

ß-Hydroxybutyrate (nmol/mL) 2.3±0.4 16.6±2.0 2.7±0.3 8.5±0.9 **<0.0001 0.0006 0.0002**

NEFA (mmol/L) 0.8±0.08 0.7±0.07 0.3±0.06 0.5±0.06 0.5822 **<0.0001 0.0231**

Cholesterol (mg/dL) 85.2±5.0 77.2±5.0 65.4±4.3 68.5±4.5 0.6121 **0.0100**  0.2898

Triglycerides (mg/dL) 21.2±4.0 13.8±4.0 25.9±3.4 24.3±3.5 0.2500 0.0584 0.4499

Glycerol (µmol/L) 135.2±28.5 79.6±13.1 53.0±8.1 82.1±15.3 0.4370 **0.0271**  **0.0197**

HDL Cholesterol (mg/dL) 42.4±2.6 36.9±2.6 29.4±2.2 35.3±2.3 0.9401 **0.0105 0.0391**

LDL Cholesterol (mg/dL) 45.4±3.0 43.6±3.0 39.4±2.6 37.2±2.8 0.5231 0.0554 0.9566

**Table S2.** Fasting metabolites in *GHR*-KO vs. wild-type pigs. Mean ±SEM; results of analysis of variance.
