# Supplementary material for: Transient juvenile hypoglycemia in GH insensitive Laron syndrome pigs is associated with insulin hypersensitivity
Source: Mol Metab. 2025 Oct 20;103:102273. doi: 10.1016/j.molmet.2025.102273 (PMC12639633; doi:10.1016/j.molmet.2025.102273)
Supplement: Multimedia component 4 [file mmc4.docx]

Parameter young WT young *GHR*-KO adult WT adult *GHR*-KO Group Age Group*Age

Cortisol (ng/mL) 16.7±4.5 32.8±4.5 16.4±3.8 19.8±4.0 **0.0364** 0.1451 0.1628

Cortisone (ng/mL) 7.2±0.7 7.1±0.7 4.9±0.6 4.9±0.6 0.9481 **0.0042** 0.9818

Corticosterone (ng/mL) 0.5±0.1 0.8±0.1 0.5±0.1 0.4±0.1 0.3126 0.3072 0.2309

Deoxycortisol (ng/mL) 0.2±0.1 3.3±0.1 0.01±0.8 0.5±0.9 0.0966 0.1659 0.2068

Deoxycorticosterone (ng/mL) 0.001±0.1 0.2±0.1 0.03±0.07 0.07±0.08 0.1965 0.4664 0.3267

Aldosterone (ng/mL) 0.05±0.01 0.07±0.01 0.03±0.01 0.05±0.01 0.1225 0.1187 0.5548

**Table S3**. Steroid hormone profile in *GHR*-KO and WT pigs. Mean ± SEM; results of analysis of variance.
