# Supplementary material for: Transient juvenile hypoglycemia in GH insensitive Laron syndrome pigs is associated with insulin hypersensitivity
Source: Mol Metab. 2025 Oct 20;103:102273. doi: 10.1016/j.molmet.2025.102273 (PMC12639633; doi:10.1016/j.molmet.2025.102273)
Supplement: Multimedia component 5 [file mmc5.docx]

Intact adult *GHR*-KO adult *GHR*-KO after early castration sex-stratified analysis

Parameter male (n=3) female (n=4) male (n=3) female (n=3) male female

Androstendione (ng/mL) 0.94±0.04 0.3±0.05 0.15±0.04 0.2±0.02 **<0.0001** 0.1312

Estradiol (ng/mL) 0.02±0.01 0.05±0.03 0.06±0.01 0.03±0.02 **0.0323**  0.5686

Testosterone (ng/mL) 4.3±0.3 0.08±0.05 0.008±0.003 0.04±0.01 **0.0012**  0.2954

Hydroxyprogesterone (ng/mL) 0.02 0.3±0.17 0.002±0.001

Progesterone (ng/mL) 0.02 15.2±8.8

**Table S4.** Sex hormones in adult intact *GHR*-KO pigs and *GHR*-KO pigs after early castration. Mean ± SEM; results of analysis of variance from student`s t test using GraphPad PRISM Version 5.04.
