# Supplementary material for: Transient juvenile hypoglycemia in GH insensitive Laron syndrome pigs is associated with insulin hypersensitivity
Source: Mol Metab. 2025 Oct 20;103:102273. doi: 10.1016/j.molmet.2025.102273 (PMC12639633; doi:10.1016/j.molmet.2025.102273)
Supplement: Multimedia component 6 [file mmc6.docx]

Parameter male WT (n=3) female WT (n=4) male *GHR*-KO (n=3) female *GHR*-KO (n=4) Sex Group*Sex

IGF1 (ng/mL) 206.3±27.3 198.8±17.8 19.7±4.2 19.3±2.2 0.8039 0.8239

Body weight (kg) 126.2±4.4 126.3±8.7 52.8±2.4 45.6±5.5 0.5912 0.5913

subcutaneous fat (cm) 0.4±0.09 0.5±0.1 1.2±0.2 1.2±0.2 0.9599 0.7285

Muscle (m. long. lumb.) (cm) 6.9±0.1 6.9±1.3 4.4±0.3 4.1±0.2 0.5436 0.6205

Glucose (mg/dL) 57.3±6.3 58.8±0.5 54.0±10.0 50.8±5.3 0.8793 0.7000

Insulin (µIU/mL) 4.9±3.0 9.2±2.2 0.6±0.3 0.5±0.2 0.2717 0.2577

ß-Hydroxybutyrate (nmol/mL) 3.1±0.4 2.5±0.4 8.6±1.2 8.3±0.8 0.5705 0.8487

NEFA (mmol/L) 0.4±0.1 0.2±0.02 0.5±0.1 0.5±0.1 0.3628 0.3355

M value (mg/kg*min) 17.6±1.8 16.9±1.5 21.3±1.9 19.9±0.6 0.4173 0.8388

Androstendione (ng/mL) 0.6±0.08 0.3±0.04 0.94±0.04 0.3±0.05 **<0.0001 0.0120**

Estradiol (ng/mL) 0.06±0.02 0.01±0.002 0.02±0.01 0.05±0.03 0.6005 **0.0320**

Testosterone (ng/mL) 1.3±0.5 0.07±0.04 4.3±0.3 0.08±0.05 **<0.0001 <0.0001**

Hydroxyprogesterone (ng/mL) 0.02 0.47±0.15 0.02 0.3±0.17

Progesterone (ng/mL) 0.02 17.3±6.7 0.02 15.2±8.8

**Table S5.** Investigation of the effects of sex in intact, adult *GHR*-KO vs. WT pigs. Mean ± SEM; results of analysis of variance.
