# Supplementary material for: Transient juvenile hypoglycemia in GH insensitive Laron syndrome pigs is associated with insulin hypersensitivity
Source: Mol Metab. 2025 Oct 20;103:102273. doi: 10.1016/j.molmet.2025.102273 (PMC12639633; doi:10.1016/j.molmet.2025.102273)
Supplement: Multimedia component 7 [file mmc7.docx]

Parameter young WT young *GHR*-KO adult WT adult *GHR*-KO Group Age Group*Age

M value (mg/kg*min) 24.1±2.9 32.2±1.6 17.2±1.1 20.5±1.4 **0.0019 <0.0001** 0.1476

Fasting HIR 9.4±1.4 0.8±0.3 12.8±3.4 0.6±1.9 **<0.0001** 0.4602 0.4033

((mg/kg*min) * (µIU/mL))

Fasting AT IR 2.2±0.4 0.2±0.06 1.7±0.4 0.3±0.3 <**0.0001** 0.4722 0.4138

((mmol/L) * (µIU/mL))

NEFA suppression (%) 91±3 95±0.4 91±2 88±3 0.8501 0.3576 0.3561

EGP_fasting_ (mg/kg*min) 3.4±0.4 2.3±0.2 1.8±0.2 1.3±0.2 **0.0007 <0.0001** 0.2021

Ra_basal_ (mg/kg*min) 3.4±0.4 2.6±0.2 1.8±1.3 1.3±0.2 **0.0013 <0.0001** 0.2015

Rd_basal_ (mg/kg*min) 3.6±0.3 2.4±0.2 1.9±0.2 1.5±0.2 **0.0029 <0.0001** 0.1004

Gluconeogenesis (%) * 55±11 82 77±18 79±9 0.2860 0.4867 0.3644

Glucose from 2.0±1.0 2.5 1.3±0.2 1.1±0.2 0.7997 0.0168 0.2756

gluconeogenesis (mg/kg*min)

**Table S6.** *In vivo* assessment of insulin sensitivity, glucose turnover and gluconeogenesis proportion in *GHR*-KO vs. WT pigs. *The proportion of gluconeogenesis and derived glucose was just assessed in young WT n=4; young *GHR*-KO n=1; adult WT n=3; adult *GHR*-KO pigs n=5. Mean ± SEM; results of analysis of variance.
