# Supplementary material for: Transient juvenile hypoglycemia in GH insensitive Laron syndrome pigs is associated with insulin hypersensitivity
Source: Mol Metab. 2025 Oct 20;103:102273. doi: 10.1016/j.molmet.2025.102273 (PMC12639633; doi:10.1016/j.molmet.2025.102273)
Supplement: Multimedia component 8 [file mmc8.docx]

Parameter young WT young *GHR*-KO adult WT adult *GHR*-KO Group Age Group*Age

Sum of ACs (µM) 0.89±0.16 1.50±0.06 1.07±0.08 1.58±0.04 **<0.0001** 0.1650 0.6145

Sum of long-chain ACs (µM) 0.09±0.03 0.15±0.01 0.02±0.01 0.10±0.02 **0.0026** **0.0084** 0.6717

∑ (C14-C18)

Sum of short-chain ACs (µM) 0.78±0.14 1.30±0.07 1.00±0.08 1.52±0.08 **<0.0001 0.0313** 0.9974

∑ (C2-C5)

CPT1 ratio 0.012±0.002 0.018±0.002 0.005±0.0006 0.0077±0.0013 **0.0097** <**0.0001** 0.2614

(C16 + C18) / C0

Beta-oxidation 0.13±0.01 0.22±0.02 0.11±0.005 0.14±0.01 **<0.0001** **0.0004**  **0.0432**

(C2 + C3) / C0

C0 (µM) 4.78±0.49 5.21±0.51 7.84±0.66 9.02±0.39 0.1549 **<0.0001** 0.4935

C2 (µM) 0.58±0.09 1.03±0.07 0.74±0.05 1.08±0.03 **<0.0001** 0.0806 0.3858

C3 (µM) 0.073±0.008 0.102±0.005 0.094±0.008 0.098±0.004 **0.0238** 0.2518 0.0903

C3-DC/ C4-OH (µM) 0.098±0.008 0.064±0.007 0.092±0.007 0.095±0.012 0.1113 0.2054 0.0634

C3-OH(µM) 0.019±0.003 0.023±0.004 0.020±0.003 0.027±0.001 0.0736 0.3385 0.4904

C4 (µM) 0.040± 0.004 0.06± 0.005 0.050± 0.009 0.069± 0.007 **0.0145**  0.1894 0.9428

C4.1 (µM) 0.042±0.002 0.035±0.001 0.039±0.002 0.041±0.002 0.2706 0.3453 **0.0493**

C5 (µM) 0.027±0.006 0.057±0.003 0.051±0.002 0.061±0.002 **<0.0001** **0.00078** **0.0131**

C5.OH/C3.DC.M (µM) 0.036±0.006 0.041±0.009 0.043±0.007 0.045±0.001 0.3788 0.2256 0.7816

C6..C4.1.DC. (µM) 0.058±0.003 0.063±0.003 0.060±0.003 0.067±0.003 0.1333 0.4145 0.8089

C6.1 (µM) 0.036±0.002 0.040±0.002 0.039±0.003 0.041±0.001 0.2677 0.3882 0.6639

C7.DC (µM) 0.015±0.002 0.020±0.001 0.018±0.002 0.013±0.001 0.9629 0.2248 **0.0123**

C8 (µM) 0.074±0.003 0.075±0.002 0.073±0.002 0.074±0.002 0.5632 0.6563 0.8786

C9 (µM) 0.014±0.001 0.015±0.002 0.015±0.001 0.015±0.003 0.7767 0.7548 0.8721

C10 (µM) 0.068±0.003 0.064±0.004 0.058±0.002 0.065±0.002 0.4955 0.1155 **0.0441**

C10.1 (µM) 0.027±0.002 0.026±0.001 0.024±0.002 0.027±0.002 0.6220 0.7544 0.5016

C10.2 (µM) 0.034±0.002 0.035±0.003 0.031±0.002 0.034±0.001 0.1952 0.1364 0.5555

C12 (µM) 0.019±0.002 0.030±0.003 0.018±0.002 0.029±0.002 **<0.0001** 0.4785 0.9369

C12.DC (µM) 0.106±0.005 0.102±0.005 0.099±0.004 0.100±0.002 0.7086 0.2346 0.3105

C12.1 (µM) 0.026±0.002 0.028±0.002 0.024±0.002 0.027±0.001 0.1556 0.5481 0.5005

C14 (µM) 0.015±0.002 0.018±0.001 0.013±0.001 0.019±0.002 **0.0045** 0.3202 0.2766

C14.1 (µM) 0.015±0.002 0.018±0.001 0.013±0.001 0.018±0.002 **0.0190** 0.5291 0.4417

C14.1.OH (µM) 0.006±0.001 0.008±0.001 0.006±0.001 0.007±0.001 0.0576 0.8975 0.6998

C14.2 (µM) 0.005±0.001 0.006±0.001 0.004±0.001 0.002±0.001 0.9280 0.3339 0.6671

C14.2.OH (µM) 0.009±0.001 0.009±0.001 0.008±0.001 0.008±0.001 0.8025 0.1921 0.9827

C16 (µM) 0.029±0.006 0.049±0.002 0.020±0.002 0.035±0.006 **0.0012**  **0.0208** 0.6195

C16.1 (µM) 0.023±0.001 0.025±0.001 0.021±0.001 0.025±0.001 **0.0002** 0.3549 0.2319

C16.2.OH (µM) 0.010±0.001 0.011±0.001 0.012±0.001 0.011±0.001 0.2326 **0.0319** 0.1585

C18 (µM) 0.027±0.005 0.041±0.002 0.016±0.002 0.027±0.005 **0.0035** **0.0031** 0.6802

C18.1 (µM) 0.033±0.005 0.041±0.001 0.018±0.002 0.030±0.005 **0.0175** **0.0038** 0.6111

C18.2 (µM) 0.010±0.002 0.011±0.001 0.008±0.001 0.009±0.001 0.3623 0.1018 0.8827

**Table S7.** Acylcarnitine (AC) profile in *GHR*-KO and WT pigs. Mean ± SEM; results of analysis of variance.
