# Supplementary material for: Transient juvenile hypoglycemia in GH insensitive Laron syndrome pigs is associated with insulin hypersensitivity
Source: Mol Metab. 2025 Oct 20;103:102273. doi: 10.1016/j.molmet.2025.102273 (PMC12639633; doi:10.1016/j.molmet.2025.102273)
Supplement: Multimedia component 9 [file mmc9.docx]

Parameter young WT young *GHR*-KO adult WT adult *GHR*-KO Group Age Group*Age

AAs (µM) 2338±126 1736±31 2466±159 2088±58.2 **0.0004**  **0.0498** 0.3425

Solely Glucogenic AAs (µM) * 1969±128 1345±30 2027±14.4 1642±54.0 **0.0001** 0.1068 0.2673

Solely Ketogenic AAs (µM) ** 81.9±12.8 112.9±2.5 92.8±9.32 119±5.65 **0.0030** 0.3273 0.7789

BCAAs (µM) 147±9.27 131±4.94 166±5.54 152±5.89 **0.0344** **0.0050** 0.8536

Non-Essential AAs (µM) 1883±118 1230±25 1911±146 1528±60.8 **<0.0001** 0.1447 0.2219

Ratio of NEAA to EAA 4.19±0.33 2.44±0.09 3.43±0.19 2.75±0.18 **<0.0001** 0.2996 **0.0177**

Alanine (µM) 318±23.9 169±12.5 276±36.9 221±8.79 **0.0007**  0.8490 0.0740

Arginine (µM) 130±9.3 116±4.4 171±4.0 126±4.42 **<0.0001** **0.0002 0.0135**

Asparagine (µM) 47.5±8.9 30.9±0.64 39.9±3.6 35.4±2.39 **0.0299** 0.7333 0.1981

Aspartic acid (µM) 12.4±1.7 7.0±0.4 12.1±1.4 8.72±0.69 **0.0012** 0.5346 0.4076

Citrulline (µM) 70.7±11.0 68.1±4.3 96.7±14.9 91.9±8.94 0.7535 **0.0432** 0.9283

Glutamine (µM) 351±37.1 150±8.3 280±19.0 190±7.14 **<0.0001** 0.4451 **0.0106**

Glutaminic acid (µM) 133±9.0 55.4±3.25 184±22.7 77.9±8.93 **<0.0001** **0.0252** 0.3623

Glycine (µM) 491±41.3 382±10.0 484±37.3 456±34.7 0.0660 0.3467 0.2595

Histidine (µM) 26.3±1.5 38.5±0.7 62.2±5.9 51.1±2.07 0.8878 **<0.0001** 0.0063

Isoleucine (µM) 15.2±1.1 12.6±0.4 15.2±0.6 13.8±0.99 **0.0307** 0.4929 0.5032

Leucine (µM) 2.45±0.17 2.20±0.11 2.76±0.14 2.35±0.18 0.0535 0.1648 0.6552

Lysine (µM) 79.4±12.9 110.7±2.5 90.0±9.4 115±7.68 **0.0051** 0.3945 0.7688

Methionine (µM) 19.2±1.3 14.9±0.6 20.9±0.9 20.8±1.07 **0.0413** **0.0015** 0.0624

Ornithine (µM) 45.3±4.48 40.4±1.49 59.9±7.17 49.9±4.89 0.1964 **0.0415**  0.6479

Phenylalanine (µM) 56.6±3.22 65.5±2.05 71.7±2.68 70.6±3.92 0.2400 **0.0058** 0.1454

Proline (µM) 124.3±7.9 75.7±3.27 145±18.2 109±11.4 **0.0051** 0.0539 0.6210

Serine (µM) 89.4±9.0 73.5±4.0 91.9±1.6 87.2±2.20 **0.0292** 0.0782 0.2182

Threonine (µM) 97.0±14.2 116±10.2 112±12.5 117±7.58 0.2909 0.4581 0.5608

Tryptophan (µM) 29.3±1.9 29.6±1.8 32.1±1.4 31.3±1.81 0.8868 0.2032 0.7601

Tyrosine (µM) 69.6±7.5 62.5±3.7 70.9±4.6 71.5±5.07 0.5529 0.3480 0.4780

Valine (µM) 129±8.2 116±4.6 148±5.0 136±4.77 **0.0408** **0.0025** 0.9033

**Table S8.** Amino acid (AA) profile in *GHR*-KO and WT pigs. Mean ± SEM; results of analysis of variance. *Solely Glucogenic AAs: Ala + Arg + Asn + Asp + Gln + Glu + Gly + His + Met + Pro + Ser + Thr + Val; **Solely Ketogenic AAs: Leu + Lys
