# Supplementary material for: Transient juvenile hypoglycemia in GH insensitive Laron syndrome pigs is associated with insulin hypersensitivity
Source: Mol Metab. 2025 Oct 20;103:102273. doi: 10.1016/j.molmet.2025.102273 (PMC12639633; doi:10.1016/j.molmet.2025.102273)
Supplement: Multimedia component 10 [file mmc10.docx]

Parameter young WT young *GHR*-KO adult WT adult *GHR*-KO Group Age Group*Age

Ac.Orn (µM) 14.2±2.81 4.98±0.66 7.72±1.51 4.86±0.82 **0.0011 0.0490** 0.0567

ADMA (µM) 0.40±0.02 0.31±0.01 0.34±0.02 0.29±0.02 **0.0019** 0.0847 0.2560

Alpha-AAA (µM) 1.02±0.33 2.20±0.22 1.74±0.42 2.29±0.28 **0.0205** 0.2485 0.3760

Creatinine (µM) 67.7±2.9 70.6±6.67 96.7±6.80 84.2±3.42 0.4197 **0.0010** 0.1888

Kynurenine (µM) 0.49±0.03 0.68±0.05 0.38±0.05 0.59±0.05 **0.0006** 0.0552 0.7336

Met-SO (µM) 1.00±0.16 1.04±0.06 1.16±0.13 1.39±0.09 0.2694 0.0422 0.4249

Putrescine (µM) 0.13±0.02 0.11±0.01 0.10±0.01 0.10±0.01 0.4166 0.1284 0.4029

Spermidine (µM) 3.99±0.53 4.93±0.43 3.03±0.29 4.67±1.16 0.1078 0.4415 0.6522

t4-OH-Pro (µM) 14.9±0.85 6.42±0.68 7.77±0.99 5.80±0.58 **<0.0001 <0.0001** **0.0008**

Taurine (µM) 52.3±3.04 56.2±2.88 48.3±1.36 61.2±2.22 **0.0018**  0.8352 0.0711

Total DMA (µM) 3.27±0.29 2.66±0.15 2.25±0.21 2.36±0.04 0.1975 **0.0022** 0.0669

**Table S9.** Profile of biogenic amines in *GHR*-KO and WT pigs. Mean ± SEM; results of analysis of variance.
