# Supplementary material for: Transient juvenile hypoglycemia in GH insensitive Laron syndrome pigs is associated with insulin hypersensitivity
Source: Mol Metab. 2025 Oct 20;103:102273. doi: 10.1016/j.molmet.2025.102273 (PMC12639633; doi:10.1016/j.molmet.2025.102273)
Supplement: Multimedia component 11 [file mmc11.docx]

Parameter young WT young *GHR*-KO adult WT adult *GHR*-KO Group Age Group*Age

Sum of MUFA LPCs (µM) 20.2±2.31 14.3±0.90 12.4±1.87 15.3±0.98 0.3722 **0.0489** **0.0134**

Sum of PUFA LPCs (µM) 38.2±2.84 25.1±1.59 24.3±3.25 25.2±1.68 **0.0290 0.0148 0.0126**

Sum of UFA LPCs (µM) 58.4±5.12 39.4±2.41 36.6±5.03 40.5±2.51 0.0807 **0.0209 0.0111**

Sum of SFA LPCs (µM) 69.7±3.46 47.9±0.57 59.6±6.52 55.1±3.71 **0.0116** 0.7624 0.0852

Ratio UFA LPC to SFA LPC 0.83±0.04 0.82±0.05 0.60±0.04 0.75±0.06 0.4486 **0.0009** 0.3155

LPC14.0 (µM) 2.19±0.37 2.43±0.44 0.97±0.06 1.40±0.37 0.3125 **0.0027**  0.7754

LPC16.0 (µM) 44.4±2.23 31.9±0.51 37.8±4.45 36.5± 2.86 0.0518 0.7653 0.1041

LPC16.1 (µM) 1.71±0.13 1.35±0.03 1.12±0.19 1.29±0.08 0.4964 **0.0304**  0.0654

LPC17.0 (µM) 1.07±0.10 0.53±0.02 0.95±0.13 0.77±0.07 **0.0016** 0.5434 0.0718

LPC18.0 (µM) 24.2±1.60 15.5±0.40 20.9±2.36 17.7±1.11 **0.0024** 0.7695 0.1240

LPC18.1 (µM) 18.5±2.20 12.9±0.88 11.2±1.71 13.9±0.90 0.3723 0.0558 **0.0132**

LPC18.2 (µM) 24.9±1.84 16.3±1.06 16.6±2.23 16.7±1.29 **0.0276**  **0.0388** **0.0241**

LPC20.3 (µM) 2.25±0.27 1.15±0.09 1.47±0.14 1.47±0.14 **0.0037**  0.1869 **0.0036**

LPC20.4 (µM) 11.1±0.88 7.59±0.53 6.17±0.93 7.05±0.65 0.1245 **0.0030** **0.0136**

LPC24.0 (µM) 0.48±0.02 0.43±0.01 0.41±0.02 0.41±0.01 0.1660 **0.0196** 0.1594

LPC26.0 (µM) 0.09±0.02 0.05±0.02 0.09±0.01 0.07±0.01 0.1282 0.7634 0.6227

**Table S10.** Lysophosphatidylcholine (LPC) profile in *GHR*-KO and WT pigs determined by targeted metabolomics. Mean ± SEM; results of analysis of variance.

MUFA, mono-unsaturated fatty acid; PUFA, poly-unsaturated fatty acid; UFA, unsaturated fatty acid; SFA, saturated fatty acid
