# Supplementary material for: Transient juvenile hypoglycemia in GH insensitive Laron syndrome pigs is associated with insulin hypersensitivity
Source: Mol Metab. 2025 Oct 20;103:102273. doi: 10.1016/j.molmet.2025.102273 (PMC12639633; doi:10.1016/j.molmet.2025.102273)
Supplement: Multimedia component 12 [file mmc12.docx]

Parameter young WT young *GHR*-KO adult WT adult *GHR*-KO Group Age Group*Age

Sum of MUFA PCs (µM) 122±15.5 104±5.61 77.9±12.6 98.4±4.83 0.8867 **0.0285** 0.0876

Sum of PUFA PCs (µM) 318±22.6 263±15.6 205±29.8 225±16.2 0.4663 **0.0043** 0.1235

Sum of UFA PCs (µM) 629±52.2 515±31.9 424±60.9 463±22.6 0.4300 **0.0122** 0.1180

Sum of SFA PCs (µM) 5.67±0.38 4.36±0.18 3.84±0.41 3.74±0.33 0.0632 **0.0029**  0.1092

Ratio of UFA PC to SFA PC 110±4.40 117±3.61 109±8.02 128±7.16 0.0710 0.5433 0.3997

PC.26.0 (µM) 0.38±0.02 0.37±0.02 0.36±0.01 0.34±0.01 0.2828 0.1186 0.6669

PC.28.1 (µM) 0.44±0.02 0.39±0.03 0.34±0.02 0.39±0.02 0.9857 0.0606 0.0748

PC.30.0 (µM) 1.42±0.07 1.27±0.06 1.10±0.08 1.10±0.07 0.3233 **0.0039**  0.3225

PC.30.2 (µM) 0.33±0.02 0.24±0.02 0.23±0.04 0.22±0.01 0.0768 **0.0379**  0.2140

PC.32.0 (µM) 3.52±0.23 2.83±0.12 2.58±0.27 2.47±0.20 0.0992 **0.0106**  0.2176

PC.32.1 (µM) 1.92±0.20 2.21±0.18 1.37±0.40 1.79±0.18 0.2288 **0.1077** 0.8397

PC.32.2 (µM) 0.72±0.05 0.60±0.04 0.51±0.06 0.54±0.04 0.3434 **0.0160** 0.1569

PC.32.3 (µM) 0.07±0.00 0.06±0.00 0.05±0.00 0.06±0.00 0.7847 **0.0067** **0.0197**

PC.34.1 (µM) 78.3±10.1 71.6±4.08 52.6±9.10 66.7±2.68 0.6142 **0.0488** 0.1669

PC.34.2 (µM) 91.7±7.33 70.5±5.58 70.3±11.0 69.0±6.78 0.2094 0.1993 0.2610

PC.34.3 (µM) 3.99±0.33 3.49±0.29 2.51±0.44 2.84±0.24 0.8094 **0.0073**  0.2515

PC.34.4 (µM) 0.26±0.02 0.25±0.02 0.17±0.02 0.19±0.01 0.7221 **0.0016** 0.2939

PC.36.0 (µM) 0.34±0.08 0.10±0.02 0.12±0.05 0.14±0.02 **0.0418**  **0.0874** **0.0101**

PC.36.1 (µM) 41.2±5.21 30.2±1.71 23.6±3.38 29.4±2.50 0.4628 **0.0137** **0.0217**

PC.36.2 (µM) 96.2±9.02 76.4±6.77 70.4±9.15 69.3±3.89 0.1823 **0.0424** 0.2290

PC.36.3 (µM) 25.9±2.56 19.0±1.42 19.2±2.86 19.7±1.10 0.1605 0.1849 0.1101

PC.36.4 (µM) 48.4±3.55 42.2±2.70 34.7±6.13 37.8±2.40 0.7300 0.0502 0.2980

PC.36.5 (µM) 3.07±0.29 2.92±0.17 2.01±0.43 2.48±0.16 0.6021 **0.0269** 0.3283

PC.36.6 (µM) 0.14±0.01 0.11±0.00 0.07±0.01 0.09±0.01 0.5309 **0.0003**  0.0223

PC.38.0 (µM) 0.34±0.03 0.25±0.02 0.19±0.01 0.18±0.01 **0.0142** **<0.0001**  **0.0249**

PC.38.1 (µM) 0.37±0.05 0.23±0.02 0.20±0.04 0.30±0.02 0.6080 0.1760 **0.0037**

PC.38.3 (µM) 27.9±2.76 18.4±1.83 20.0±1.75 19.1±1.46 **0.0147** 0.0817 **0.0369**

PC.38.4 (µM) 122±8.49 98.5±6.22 73.5±10.9 83.9±8.57 0.4922 **0.0031** 0.0852

PC.38.5 (µM) 29.0±1.98 28.1±1.51 19.5±3.17 23.7±1.86 0.5068 **0.0099** 0.3030

PC.38.6 (µM) 14.6±2.15 11.5±0.16 6.71±1.39 7.91±0.93 0.4817 **0.0004**  0.1219

PC.40.1 (µM) 0.21±0.01 0.19±0.00 0.19±0.00 0.19±0.00 0.1653 **0.0245**  **0.0288**

PC.40.2 (µM) 0.14±0.01 0.18±0.01 0.10±0.01 0.15±0.01 **0.0004** **0.0112**  0.3948

PC.40.3 (µM) 0.27±0.02 0.28±0.02 0.17±0.01 0.27±0.02 **0.0118**  **0.0180** **0.0184**

PC.40.4 (µM) 4.75±0.34 7.55±0.73 4.23±0.49 5.26±0.51 **0.0021** **0.0178** 0.1182

PC.40.5 (µM) 20.2±1.80 19.9±1.76 15.9±2.08 14.8±1.37 0.7248 **0.0185**  0.8219

PC.40.6 (µM) 16.5±2.34 10.0±0.31 6.31±1.16 6.73±0.99 **0.0372** **<0.0001** **0.0191**

PC.42.0 (µM) 0.07±0.00 0.06±0.00 0.05±0.00 0.05±0.00 0.4191 **0.0036** 0.4650

PC.42.1 (µM) 0.07±0.00 0.05±0.00 0.05±0.00 0.05±0.00 **0.0066** **0.0028** 0.1328

PC.42.2 (µM) 0.12±0.01 0.10±0.00 0.08±0.01 0.09±0.00 0.9819 **0.0011** 0.0144

PC.42.4 (µM) 0.11±0.01 0.10±0.00 0.07±0.01 0.07±0.00 0.9764 **0.0001**  0.1149

PC.42.5 (µM) 0.20±0.03 0.20±0.02 0.09±0.01 0.14±0.02 0.3045 **0.0006**  0.1992

PC.42.6 (µM) 0.31±0.06 0.34±0.02 0.15±0.02 0.24±0.03 0.0914 **0.0013** 0.3755

PC.O.28.0 (µM) 0.19±0.01 0.16±0.01 0.15±0.01 0.16±0.01 0.3405 **0.0792**  0.0805

PC.O.28.1 (µM) 0.16±0.03 0.15±0.02 0.14±0.01 0.14±0.01 0.5836 0.2999 0.6375

PC.O.30.0 (µM) 0.18±0.01 0.18±0.01 0.12±0.01 0.13±0.00 0.2601 **<0.0001** 0.3100

PC.O.30.2 (µM) 0.16±0.01 0.15±0.00 0.15±0.00 0.15±0.00 **0.0150**  0.4254 0.1508

PC.O.32.1 (µM) 0.66±0.03 0.63±0.03 0.44±0.04 0.52±0.02 0.4095 **0.0002**  0.1258

PC.O.32.2 (µM) 0.18±0.01 0.14±0.01 0.12±0.01 0.12±0.00 **0.0934** **0.0002**  0.0208

PC.O.34.0 (µM) 0.39±0.02 0.31±0.02 0.28±0.02 0.28±0.02 0.0697 **0.0026**  0.0509

PC.O.34.1 (µM) 3.74±0.21 3.02±0.15 2.47±0.30 2.91±0.16 0.5634 **0.0076** **0.0201**

PC.O.34.2 (µM) 3.00±0.11 3.09±0.18 2.50±0.25 3.21±0.18 0.0634 0.3712 0.1375

PC.O.34.3 (µM) 1.21±0.08 1.48±0.06 1.25±0.14 1.97±0.19 **0.0030** 0.0864 0.1392

PC.O.36.0 (µM) 0.11±0.01 0.10±0.01 0.09±0.01 0.09±0.00 0.8432 0.2170 0.6995

PC.O.36.1 (µM) 3.36±0.57 1.77±0.08 1.96±0.24 2.19±0.15 **0.0317** 0.1106 **0.0055**

PC.O.36.2 (µM) 5.37±0.47 3.27±0.18 3.75±0.38 3.74±0.27 **0.0068** 0.1181 **0.0068**

PC.O.36.3 (µM) 2.37±0.09 1.88±0.13 1.77±0.19 1.96±0.09 0.3237 0.0929 **0.0284**

PC.O.36.4 (µM) 2.31±0.14 2.38±0.11 1.88±0.17 1.99±0.14 0.5742 **0.0151** 0.8790

PC.O.36.5 (µM) 1.33±0.09 1.38±0.05 1.13±0.14 1.29±0.10 0.3407 0.1975 0.5974

PC.O.38.0 (µM) 0.49±0.05 0.32±0.01 0.27±0.05 0.29±0.02 0.0794 **0.0049 0.0355**

PC.O.38.1 (µM) 0.37±0.05 0.21±0.02 0.20±0.03 0.22±0.01 **0.0252 0.0099 0.0099**

PC.O.38.2 (µM) 0.91±0.07 0.56±0.04 0.64±0.05 0.63±0.03 **0.0016** 0.0517 **0.0023**

PC.O.38.3 (µM) 1.29±0.09 0.95±0.06 0.91±0.05 0.95±0.04 **0.0195 0.0045 0.0047**

PC.O.38.4 (µM) 5.41±0.27 4.45±0.21 3.64±0.31 3.92±0.29 0.2672 **0.0010**  0.0497

PC.O.38.5 (µM) 3.13±0.18 2.84±0.16 2.12±0.20 2.26±0.15 0.6877 **0.0003**  0.2516

PC.O.38.6 (µM) 0.64±0.05 0.59±0.04 0.44±0.04 0.45±0.03 0.6127 **0.0003**  0.4287

PC.O.40.1 (µM) 0.44±0.06 0.40±0.03 0.39±0.09 0.33±0.03 0.4445 0.3627 0.9510

PC.O.40.2 (µM) 0.32±0.01 0.26±0.01 0.25±0.01 0.25±0.01 **0.0262 0.0010 0.0028**

PC.O.40.3 (µM) 0.30±0.01 0.28±0.03 0.21±0.01 0.23±0.01 0.6418 **0.0006**  0.2350

PC.O.40.4 (µM) 1.12±0.05 0.98±0.06 0.78±0.06 0.84±0.05 0.5471 **0.0006**  0.0910

PC.O.40.5 (µM) 1.54±0.07 1.25±0.08 1.08±0.09 1.04±0.07 0.0582 **0.0005**  0.1376

PC.O.40.6 (µM) 1.01±0.07 0.69±0.02 0.53±0.05 0.51±0.05 **0.0046 <0.0001 0.0101**

PC.O.42.0 (µM) 0.25±0.01 0.22±0.01 0.22±0.01 0.21±0.00 **0.0171 0.0273** 0.0941

PC.O.42.1 (µM) 0.24±0.02 0.21±0.02 0.23±0.04 0.20±0.01 0.2863 0.7340 0.9534

PC.O.42.2 (µM) 0.19±0.02 0.18±0.02 0.21±0.04 0.16±0.01 0.2846 0.8928 0.6357

PC.O.42.3 (µM) 0.12±0.01 0.12±0.01 0.09±0.01 0.09±0.00 0.8193 **0.0073** 0.5005

PC.O.42.4 (µM) 0.13±0.01 0.12±0.01 0.08±0.01 0.08±0.00 0.9234 **<0.0001** 0.5053

PC.O.42.5 (µM) 0.71±0.01 0.67±0.02 0.62±0.01 0.60±0.01 0.0409 **<0.0001** 0.3514

PC.O.44.3 (µM) 0.06±0.00 0.06±0.00 0.05±0.01 0.04±0.00 0.6789 0.1680 0.7890

PC.O.44.4 (µM) 0.08±0.00 0.07±0.00 0.06±0.01 0.06±0.00 0.1891 **0.0084** 0.6187

PC.O.44.5 (µM) 0.13±0.01 0.11±0.01 0.09±0.01 0.08±0.00 0.0669 **<0.0001** 0.4186

PC.O.44.6 (µM) 0.20±0.01 0.17±0.01 0.14±0.00 0.14±0.00 **0.0343**  **<0.0001** **0.0203**

**Table S11.** Phosphatidylcholine (PC) profile in *GHR*-KO and WT pigs determined by targeted metabolomics. Mean ± SEM; results of analysis of variance.

MUFA, mono-unsaturated fatty acid; PUFA, poly-unsaturated fatty acid; UFA, unsaturated fatty acid; SFA, saturated fatty acid
