# Supplementary material for: Transient juvenile hypoglycemia in GH insensitive Laron syndrome pigs is associated with insulin hypersensitivity
Source: Mol Metab. 2025 Oct 20;103:102273. doi: 10.1016/j.molmet.2025.102273 (PMC12639633; doi:10.1016/j.molmet.2025.102273)
Supplement: Multimedia component 13 [file mmc13.docx]

Parameter young WT young *GHR*-KO adult WT adult *GHR*-KO Group Age Group*Age

SM.33.1 (µM) 1.24±0.07 1.15±0.07 1.16±0.06 1.30±0.06 0.6594 0.6310 0.0817

SM.34.1 (µM) 62.5±3.32 51.7±2.16 48.8±2.70 51.2±3.13 0.1778 **0.0274** **0.0385**

SM.34.2 (µM) 5.95±0.31 4.26±0.19 4.35±0.37 4.27±0.30 **0.0129** **0.0231** **0.0212**

SM.35.1 (µM) 2.31±0.16 1.52±0.11 1.86±0.13 1.69±0.09 **0.0014** 0.2952 **0.0247**

SM.36.1 (µM) 13.1±0.78 12.2±0.55 13.1±0.65 13.08±1.28 0.6271 0.6041 0.6698

SM.36.2 (µM) 4.02±0.21 3.34±0.14 3.51±0.21 3.42±0.29 0.1300 0.4010 0.2269

SM.38.3 (µM) 0.33±0.02 0.26±0.01 0.27±0.02 0.27±0.01 0.0610 0.1849 **0.0379**

SM.40.4 (µM) 0.63±0.07 0.51±0.04 0.50±0.08 0.54±0.03 0.5506 0.4536 0.1693

SM.41.1 (µM) 4.17±0.16 2.77±0.11 3.49±0.17 3.01±0.17 **<0.0001** 0.1946 **0.0115**

SM.41.2 (µM) 1.55±0.03 1.03±0.06 1.43±0.08 1.19±0.05 **<0.0001** 0.8478 **0.0322**

SM.42.1 (µM) 15.2±0.66 10.5±0.43 12.1±0.37 11.2±0.79 **0.0002**  0.0701 **0.0069**

SM.42.2 (µM) 14.5±0.78 10.7±1.09 14.2±0.73 12.03±0.49 **0.0008** 0.4950 0.2908

SM.43.1 (µM) 0.59±0.02 0.46±0.02 0.45±0.02 0.47±0.04 0.1153 0.0656 **0.0275**

SM.44.1 (µM) 0.10±0.01 0.08±0.01 0.07±0.01 0.09±0.01 0.5929 0.3018 **0.0188**

SM.44.2 (µM) 0.31±0.02 0.25±0.01 0.24±0.01 0.23±0.03 0.0877 **0.0322** 0.1749

**Table S12.** Sphingolipid profile in *GHR*-KO and WT pigs determined by targeted metabolomics. Mean ± SEM; results of analysis of variance.
