# Supplementary material for: Transient juvenile hypoglycemia in GH insensitive Laron syndrome pigs is associated with insulin hypersensitivity
Source: Mol Metab. 2025 Oct 20;103:102273. doi: 10.1016/j.molmet.2025.102273 (PMC12639633; doi:10.1016/j.molmet.2025.102273)
Supplement: Multimedia component 14 [file mmc14.docx]

Transcript young WT young *GHR*-KO adult WT adult *GHR*-KO Group Age Group*Age

*PPARG* 0.8±0.1 0.9±0.1 1.0±0.1 0.8±0.03 0.6490 0.3021 **0.0853**

*FASN* 0.8±0.3 0.8±0.3 1.1±0.4 1.2±0.3 0.8588 0.3232 0.7983

*SCD* 0.9±0.2 0.9±0.3 1.3±0.5 1.3±0.3 0.9275 0.2555 0.9801

*ME1* 0.8±0.2 0.9±0.3 0.8±0.3 0.9±0.2 0.6450 0.6598 0.9491

*ACACA* 0.9±0.2 0.8±0.2 1.6±0.7 1.8±0.5 0.8917 **0.0463**  0.7460

*ELOVL6* 1.2±0.3 1.3±0.3 1.6±0.5 1.4±0.2 0.9314 0.3155 0.8988

*ACSL1*  0.7±0.1 0.5±0.1 1.0±0.4 0.6±0.2 0.3292 0.1799 0.7081

*PNPLA2* 0.7±0.1 0.5±0.1 0.6±0.1 0.5±0.1 0.1538 0.1618 0.6308

*ACLY1* 1.6±0.7 1.0±0.4 4.0±2.6 3.4±1.5 0.8951 **0.0312** 0.9377

*THRSP* 0.7±0.2 0.7±0.2 1.4±0.6 1.3±0.4 0.8309 **0.0513** 0.9008

*PLIN1* 0.7±0.1 0.4±0.1 0.8±0.2 0.6±0.1 0.1803 0.6561 0.7198

*LIPE* 0.8±0.1 0.7±0.2 0.7±0.2 0.7±0.1 0.6061 0.5421 0.6298

*INSR* 0.8±0.1 1.0±0.04 0.9±0.1 0.9±0.1 0.8237 0.2848 0.4612

*IGF1R*  0.8±0.1 0.8±0.1 0.7±0.1 0.7±0.1 0.8477 **0.0734** 0.7051

*GLUT4* 0.6±0.1 0.6±0.2 1.2±0.4 0.7±0.2 0.3225 0.1488 0.4356

*GLUT1* 0.5±0.1 0.2±0.03 0.4±0.1 0.2±0.03 **0.0504** 0.6435 0.6668

*IL1B* 1.9±0.7 4.0±1.1 3.2±1.6 9.6±2.9 **0.0738** 0.4009 0.3214

*IL6* 2.4±0.9 2.7±0.7 3.8±1.8 3.3±0.6 0.5431 0.4735 0.2804

*ADIPOQ* 0.9±0.1 0.8±0.1 0.8±0.1 0.8±0.1 0.5048 0.5877 0.3870

*MCP1* 1.3±0.3 1.6±0.2 0.9±0.2 2.2±0.4 **0.0060**  0.9136 0.5728

*ADRB3* 1.2±0.3 1.6±0.3 1.0±0.3 2.2±1.1 0.4299 0.4948 0.3952

*NPR3* 1.1±0.2 2.3±0.3 1.2±0.2 3.6±0.3 **<0.0001** 0.1727 **0.0587**

**Table S13.** qPCR analysis of relative abundance of genes involved in lipolysis, adipogenesis and lipid desaturation in *GHR*-KO vs. WT subcutaneous fat. Mean ± SEM; results of analysis of variance.

PPARG, Peroxisome proliferator-activated receptor gamma; FASN. Fatty acid synthase; SCD, Stearoyl-CoA desaturase; ME1, NADP-dependent malic enzyme; ACACA, Acetyl-CoA carboxylase 1; ELOVL6, Very long chain fatty acid elongase 6; ACSL1, Long-chain-fatty-acid--CoA ligase 1; PNPLA2, Patatin-like phospholipase domain-containing protein 2; ACLY, ATP-citrate synthase; THRSP, Thyroid hormone-inducible hepatic protein; PLIN1, Perilipin-1; LIPE, Hormone-sensitive lipase; INSR, Insulin receptor; IGF1R, Insulin-like growth factor 1 receptor; GLUT4, Solute carrier family 2, facilitated glucose transporter member 4; GLUT1, Solute carrier family 2, facilitated glucose transporter member 1; IL1B, Interleukin-1 beta; IL6, Interleukin-6; ADIPOQ, Adiponectin; MCP1, Monocyte chemoattractant protein-1; ADRB3, Beta-3 adrenergic receptor;, NPR3, Natriuretic Peptide Receptor-3
